# TRAIL^+^ monocytes and monocyte-related cells cause lung damage and thereby increase susceptibility to influenza–*Streptococcus pneumoniae* coinfection

**DOI:** 10.15252/embr.201540473

**Published:** 2015-08-18

**Authors:** Gregory T Ellis, Sophia Davidson, Stefania Crotta, Nora Branzk, Venizelos Papayannopoulos, Andreas Wack

**Affiliations:** Mill Hill Laboratory, Francis Crick InstituteLondon, UK

**Keywords:** C-C-chemokine receptor type (CCR) 2, influenza, neutrophil, *Streptococcus pneumoniae*, TNF-related apoptosis-inducing ligand (TRAIL)

## Abstract

*Streptococcus pneumoniae* coinfection is a major cause of influenza-associated mortality; however, the mechanisms underlying pathogenesis or protection remain unclear. Using a clinically relevant mouse model, we identify immune-mediated damage early during coinfection as a new mechanism causing susceptibility. Coinfected CCR2^−/−^ mice lacking monocytes and monocyte-derived cells control bacterial invasion better, show reduced epithelial damage and are overall more resistant than wild-type controls. In influenza-infected wild-type lungs, monocytes and monocyte-derived cells are the major cell populations expressing the apoptosis-inducing ligand TRAIL. Accordingly, anti-TRAIL treatment reduces bacterial load and protects against coinfection if administered during viral infection, but not following bacterial exposure. Post-influenza bacterial outgrowth induces a strong proinflammatory cytokine response and massive inflammatory cell infiltrate. Depletion of neutrophils or blockade of TNF-α facilitate bacterial outgrowth, leading to increased mortality, demonstrating that these factors aid bacterial control. We conclude that inflammatory monocytes recruited early, during the viral phase of coinfection, induce TRAIL-mediated lung damage, which facilitates bacterial invasion, while TNF-α and neutrophil responses help control subsequent bacterial outgrowth. We thus identify novel determinants of protection versus pathology in influenza–*Streptococcus pneumoniae* coinfection.

## Introduction

Influenza A virus (IAV) is a major human respiratory pathogen 1, and deaths after influenza infections are frequently due to complications associated with secondary bacterial infections. These are caused by *Streptococcus pneumoniae* (*S. pneumoniae*, *Strep*), *Staphylococcus aureus* and other bacteria that often colonize the upper respiratory tract resulting in asymptomatic carriage but can also lead to pneumonia and septicaemia 2-4. Among these, *S. pneumoniae* was the most commonly detected coinfection in both fatal cases of the 1918 Spanish influenza pandemic 5 and hospitalized patients in the recent 2009 swine influenza pandemic 6. Furthermore, a link between seasonal influenza and invasive pneumococcal pneumonia has been described 7, 8. Together, these results indicate that bacterial coinfection is a significant factor of the influenza-related public health burden.

Investigation of acute coinfection in mouse models has identified a range of possible mechanisms for IAV–*S. pneumoniae* coinfection 9, suggesting that multiple factors contribute to bacterial susceptibility. The majority of previous studies typically investigated two broad factors: direct viral-mediated lung damage allowing increased bacterial colonization, or impairment of the antibacterial immune response. In this study, we use a clinically relevant disease setting where the relative importance of these previously described factors is decreased. We uncover a new mechanism of coinfection: immune damage caused by the response to mild influenza allowing bacterial colonization.

Acute coinfection models are characterized by loss of bacterial control in the lung and bacterial dissemination 10, increases in many proinflammatory immune cells and cytokines 11 and in some models of severe viral infections, prolonged viral presence 12. Although a strong immune response is frequently observed, many studies have reported prior influenza impairs the antibacterial response. Components of the antiviral response, such as type I or type II IFN, have also been identified as potentially promoting disease in coinfection 13, 14, 15, 16, 17. Other suggested disease-promoting effects in coinfection are depletion of alveolar macrophages by influenza virus infection 18 and impairment of their function by inhibitory receptors expressed on apoptotic cells 10. However, for many functions in the immune response, it is still unclear whether they are impaired and whether they are protective or pathogenic during IAV–*S. pneumoniae* coinfection.

Lung damage and changes in physiological state directly caused by influenza virus have also been implicated as promoting coinfection, typically using highly pathogenic viral strains. The viral cytotoxic peptide PB1-F2 promotes susceptibility to secondary infection 19, and influenza can directly promote bacterial colonization by reducing ciliary beating 20 and increasing sialic acid availability 21.

Inflammatory monocytes are among the most abundant cells to be recruited into coinfected lungs, but their role in coinfection has not been addressed. Release of inflammatory monocytes from the bone marrow into blood and recruitment into peripheral organs are dependent on C-C-chemokine receptor type 2 (CCR2) 22. In *S. pneumoniae* infection, a protective role of inflammatory monocytes was shown in CCR2^−/−^ mice and by overexpression of the CCR2 ligand MCP-1 23, 24. Inflammatory monocytes have been associated with lung damage in severe influenza infection, as CCR2^−/−^ mice have increased lung integrity and greater disease resistance 25, 26, but inflammatory monocytes are required for full adaptive anti-influenza responses 27. Given these findings, the role of inflammatory monocytes during IAV–*S. pneumoniae* coinfection is difficult to predict and merits investigation.

TNF-related apoptosis-inducing ligand (TRAIL) is a cell-death-inducing ligand that mediates apoptosis of target cells in mice through the engagement of its cellular receptor death receptor 5 (DR5) 28. Similar to inflammatory monocytes, the effects of TRAIL are variable in single infections and have not been studied in IAV–*S. pneumoniae* coinfection. Studies in single *S. pneumoniae* infection 29 show that TRAIL contributes to protection, while severe influenza infection is associated with high frequencies of TRAIL-expressing inflammatory monocytes and damage to the infected lung epithelia 25, 30. In contrast, other studies show that TRAIL contributes to protection in comparably milder influenza infection 31. How TRAIL-dependent mechanisms affect the outcome of coinfection remains to be determined.

In addition to an incomplete understanding of the upstream factors promoting bacterial invasion, it is also still unclear whether aspects of the strong inflammatory immune response post-bacterial exposure, such as neutrophils or TNF-α, are protective or pathogenic. Neutrophils are potent innate effector cells with known antibacterial functions and a high potential for tissue damage 32, and their numbers are massively increased in coinfected lungs. Reduced neutrophil action was proposed to contribute to susceptibility, as production of reactive oxygen species and phagocytosis by lung neutrophils were reduced in influenza infection 33. Similarly, neutrophil apoptosis was found to be increased by *in vitro* exposure to IAV and *S. pneumoniae*, and neutrophil ROS production was suggested to contribute to this 34.

Despite the suggestion that they are impaired, a protective role of neutrophils in coinfection was also indirectly implied in studies of mice deficient for the type I IFN receptor. Compared to wild-type controls, these mice have a stronger neutrophil response and reduced susceptibility to coinfection 14; however, coinfection in neutrophil-depleted mice was not performed. Where neutrophil depletion was attempted, the coinfection was so severe that no margin to observe further aggravation was left 35. Another study also attempted to deplete neutrophils in coinfection—however, antibodies specific for Gr-1 were used, which deplete plasmacytoid dentric cells (pDCs), monocytes and neutrophils and thus render interpretation of results difficult 33. Given the massive recruitment of neutrophils into coinfected lungs, they may aid protection by bacterial control, and impairment of their function by prior influenza may be a contributing factor to coinfection. Alternatively, these cells may be a harmful downstream source of immunopathology. Therefore, the role of neutrophils in coinfection remains unclear.

TNF-α is a proinflammatory cytokine massively induced in coinfected lungs 11, 36, but its role in coinfection has not been directly addressed. TNF-α protects in single infection with *S. pneumoniae* 37, but its role in influenza is less clear: in severe infection, TNF-α contributes to pathology, while in mild influenza, it is protective 38, 39, 40. Therefore, the net effect of TNF-α in coinfection is difficult to predict and has not yet been investigated.

In this study, we dissect the early and late immune response to coinfection and propose a novel immune-mediated mechanism leading to increased mortality in IAV–*S. pneumoniae* coinfection. We find that upstream of bacterial colonization, inflammatory monocytes and other CCR2-dependent myeloid cells recruited during influenza infection promote susceptibility by compromising lung integrity through a TRAIL-mediated mechanism. We then profile the response to subsequent bacterial infection and observe massive increases in both neutrophils and TNF-α. We find no functional impairment of neutrophils in coinfection and show that both neutrophils and TNF-α are essential for controlling bacterial invasion and dissemination once colonization has occurred. This distinction between pathogenic and protective immune mechanisms will help inform treatment options in coinfection.

## Results

### Influenza predisposes mice to *Streptococcus pneumoniae* coinfection leading to bacterial outgrowth and a strong immune response

**Figure 1:**
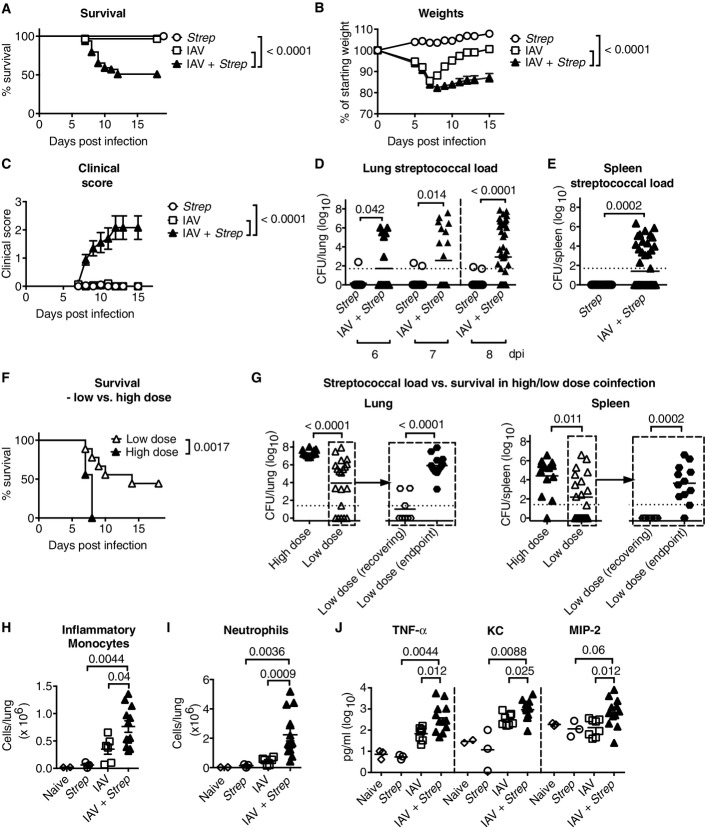
Influenza A predisposes mice to *Streptococcus pneumoniae* coinfection A–C Mortality (A), weights (B) and clinical scores (C) following intranasal infection of C57BL/6 mice with 400 TCID_50_/30 μl IAV X31, 2 × 10^5^ CFU/30 μl *S. pneumoniae* D39 or mock (PBS) (data shown are pooled from five independent experiments, *n* = 6–12; this dosing regimen hereafter referred to as “low dose”; for clarity, means shown include euthanized mice at endpoint weight or clinical score).

In our model of coinfection, we used common, low-pathogenicity laboratory strains of IAV (X31) and *S. pneumoniae* (D39). We performed secondary *S. pneumoniae* infection at 5 days post-infection (dpi), which was established as the time point of maximum synergy 41. Pathogen doses were chosen to cause almost no mortality in single infections and 50% mortality in coinfection (Fig1A), to allow the assessment of protective and deleterious effects of intervention. IAV induced transient weight loss but no clinical signs, while *S. pneumoniae* alone caused no weight loss or clinical signs (Fig1B and C). In contrast, coinfection with both pathogens caused mortality rapidly after bacterial infection on day 5 (Fig1A), with substantial weight loss and elevated clinical scores. Mild to moderate single infections therefore allowed us to study coinfection synergy, which results in severe disease.

D, E Pneumococcal load in the lung (D) from 6 to 8 dpi and in the spleen (E) at 8 dpi during low-dose coinfection (6–7 dpi data shown are pooled from four independent experiments, 8 dpi data (after dashed line) are pooled from eight independent experiments, and dotted line indicates detection limit, *n* = 3–10).

F Mortality following coinfection with high (8 × 10^3^ TCID_50_ IAV, 2 × 10^7^ CFU *S. pneumoniae*) or low (as above) coinfection doses (*n* = 9).

G Comparison of lung pneumococcal load in mice harvested upon reaching endpoint or concurrently harvested recovering (gaining weight) mice, at low and high dose from 8 to 10 dpi. All mice at high dose reached endpoint; all low-dose mice are grouped (left panels) and then separated into recovering and endpoint groups (right panels) (dotted line indicates detection limit, *n* = 13–21).

H, I Quantification of inflammatory monocytes (H) or neutrophils (I) at 7 dpi by flow cytometry during low-dose coinfection (data shown are pooled from two independent experiments; *n* = 2–6).

J Multiplex quantification of TNF-α, KC and MIP2 in the airways at 7 dpi during low-dose coinfection (data shown are pooled from two independent experiments, *n* = 2–6). Data information: Data are displayed as percentage survival (mortality), geometric means (pneumococcal loads) or arithmetic means ± SEM (weights, clinical scores, cells and cytokines). Significance was assessed by log-rank (Mantel–Cox) test (mortality), two-way ANOVA (weights and clinical scores) or Mann–Whitney *U*-test (pneumococcal loads, cells and cytokines).

To determine the causes of mortality in coinfection, we profiled bacterial loads. In the single bacterial infection, *S. pneumoniae* was infrequently detected in the lung or spleen in the first 3 days following infection. In contrast, during coinfection, a wide spread of pneumococcal loads were detected on days 6–8 (Fig1D), with many mice having high bacterial load and other mice being free of bacteria. Similarly, systemic spread was found in about 50% of coinfected mice (Fig1E).

To understand whether high bacterial load and systemic spread are predictors of mortality in our low-dose 50% mortality model, we first compared our results with a high-dose coinfection regimen (FigEV1A–C). Here, almost all coinfected mice died and had high bacterial loads in the lung and systemically, while singly infected mice controlled bacterial load, showed < 10% mortality and only transient clinical signs such as piloerection, hunched posture and laboured breathing (FigEV1C–E). In contrast to bacterial load, virus titres were unaffected by the bacterial superinfection, and no systemic viral spread was observed (FigEV1F and G). We compared high-dose and low-dose coinfection directly in an experiment where mortality and bacterial control was assessed (Fig1F). We harvested organs from mice as they reached clinical endpoint (this occurred from 8 to 10 dpi). As at low dose, not all mice reach endpoint, we concurrently harvested recovering mice (i.e. mice gaining weight) as a control group. When we profiled pneumococcal loads, we found a strong correlation between mice reaching clinical endpoint and high pneumococcal load in the lung and spleen (Fig1G). Low-dose coinfection therefore allowed us to confirm directly that bacterial outgrowth correlated with mortality in coinfection, and the wide spread of bacterial load (Fig1D and E) reflects the ability of about half of the mice to control the bacterial infection, while the other half succumbs to coinfection. Supporting this, we find infection with live bacteria is required for synergy in mortality during coinfection, as treatment with heat-killed streptococci or TLR2 agonist did not reproduce this effect (Fig EV1H).

**Figure:**
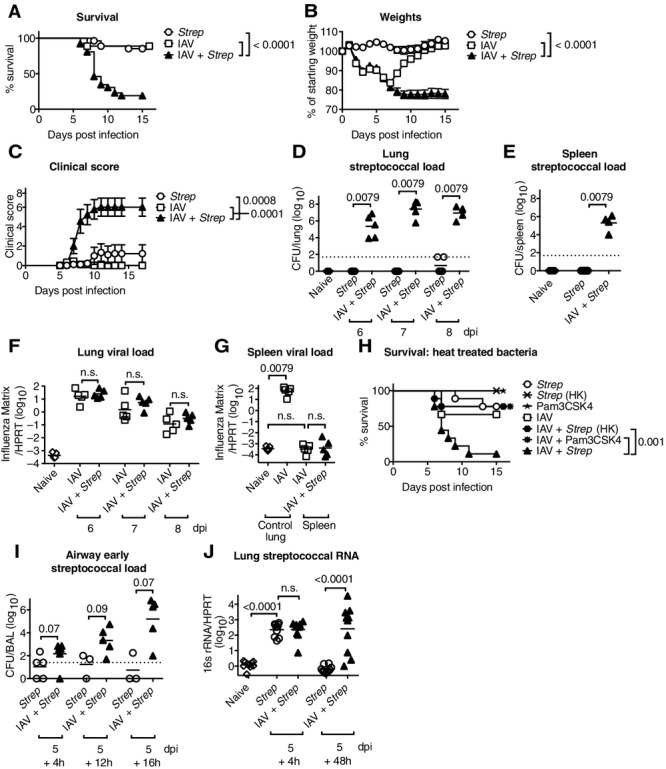
Mortality in coinfection is linked to outgrowth of live bacteria A–C Mortality (A), weights (B) and clinical scores (C) following infection with 8 × 10^3^ TCID_50_/30 μl IAV X31, 2 × 10^7^ CFU/30 μl *S. pneumoniae* D39 or mock (PBS) (data shown are pooled from four independent experiments, *n* = 6–9; this dosing regimen hereafter referred to as “high dose”; for clarity, means shown include euthanized mice at endpoint weight or clinical score).

D, E Pneumococcal load in the lung (D) from 6 to 8 dpi and in the spleen (E) at 8 dpi during low-dose coinfection (dotted line indicates detection limit, *n* = 4–5).

F Quantitative PCR for influenza matrix RNA in the lung during high-dose coinfection from 6 to 8 dpi (*n* = 5).

G Quantitative PCR for influenza matrix RNA in the spleen at 8 dpi during high-dose coinfection, compared to influenza-infected positive control lung (6 dpi 8 × 10^3^ TCID_50_ IAV) (*n* = 5).

H Mortality following high-dose coinfection with live *S. pneumoniae*, heat-killed *S. pneumoniae* or Pam3CSK4 (15 μg) (representative of two independent experiments, *n* = 6–9).

I Pneumococcal load in the airways early during high-dose coinfection from 5 dpi + 4 h to 5 dpi + 16 h (dotted line indicates detection limit, *n* = 5).

J Quantitative PCR for pneumococcal 16 s rRNA in the lung during high-dose coinfection from 5 dpi+4 h to 5 dpi + 48 h (*n* = 10). Data information: Data are displayed as percentage survival (mortality), geometric means (viral and bacterial loads, bacterial RNA) or arithmetic means ± SEM (weights and clinical scores). Significance was assessed by Mann–Whitney *U*-test (viral and bacterial loads, bacterial RNA), two-way ANOVA (weights and clinical scores) or log-rank (Mantel–Cox) test (mortality). n.s. = not significant.

Taking advantage of the homogeneity of data within groups in the high-dose regimen, we assessed bacterial loads at early time points after exposure (FigEV1I). Both in single and in coinfection, *S. pneumoniae* loads were strongly reduced, compared to bacterial inoculate, at 4 h post-infection. In coinfected mice, bacterial load then consistently started to rise over time, while pneumococcus growth remained low in singly infected mice, leading to detectable differences as early as 12 h post-infection (FigEV1I). We verified bacterial inoculation by quantifying bacterial RNA in the lung at 4 and 48 h post-infection; this detects both live and dead bacteria. High levels of bacterial RNA were detected in both singly infected and coinfected mice at 4 h, indicating successful inoculation. Consistent with the rapid bacterial clearance observed in singly infected mice, pneumococcal RNA was only detected in coinfected mice at 48 h (FigEV1J).

To profile the immune response induced by coinfection, we compared cell infiltrates into singly infected and coinfected lungs at day 7 after IAV infection (2 days after subsequent bacterial infection given at 5 dpi). Cell populations were quantified by flow cytometry (Fig1H and I for low-dose coinfection, Fig EV2A–C for high-dose coinfection). The most strongly increased cell populations compared to single infections were inflammatory monocytes and neutrophils (Figs1H and I, and EV2A and B). In contrast, CD4, CD8 T cells and NK cells were similar to IAV infection, and both coinfection and influenza caused a modest but non-significant reduction in alveolar macrophages (FigEV2C). Histology showed little immune cell infiltrate in *S. pneumoniae*-infected mice, a modest infiltrate during influenza and massive cellular recruitment in coinfected mice, with few unobstructed airspaces (FigEV2D).

**Figure:**
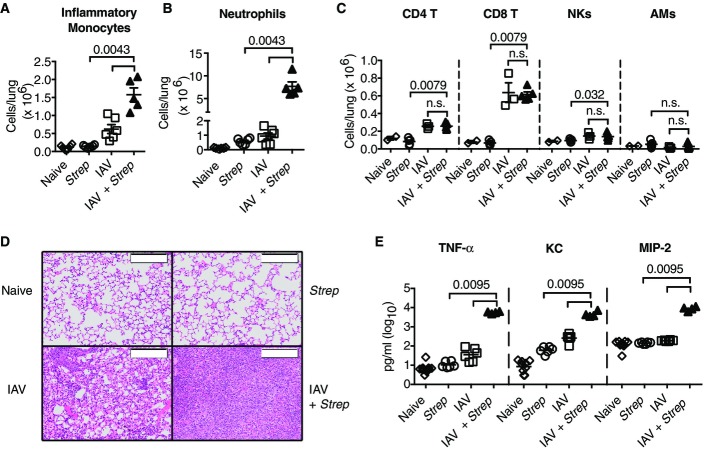
Quantification of cells and cytokines during high-dose coinfection A, B Quantification of inflammatory monocytes (A) and neutrophils (B) during high-dose coinfection at 7 dpi by flow cytometry (data shown are pooled from two independent experiments, *n* = 2–3).

C Quantification of CD4 T cells (CD3^+^CD4^+^), CD8 T cells (CD3^+^CD8^+^), NK cells (CD3^−^CD4^−^CD8^−^NKp46^+^) and alveolar macrophages during high-dose coinfection at 7 dpi (*n* = 2–5).

D H&E staining of lung tissue sections at 8 dpi during high-dose coinfection. Scale bar indicates 200 μm (*n* = 2–3).

E Multiplex quantification of TNF-α, KC and MIP2 in the airways at 7 dpi during high-dose coinfection (*n* = 2–6). Data information: Data are displayed as arithmetic means ± SEM. Significance was assessed by Mann–Whitney *U*-test (viral and bacterial loads, bacterial RNA). n.s. = not significant.

We also profiled the cytokine response. Coinfection caused a large increase in TNF-α, which was largely absent in both single infections (Fig1J). Consistent with the large neutrophil response observed, we also found a rise in neutrophil-recruiting chemokines KC and MIP-2. While cytokine amounts show more spread in low-dose coinfection with 50% mortality (Fig1J), more consistently high cytokine values were found with the high-dose regimen (Fig EV2E). Overall, the magnitude of changes is lower and the spread of data is greater in low-dose coinfection than in high-dose coinfection, but we found all parameters change in the same direction when comparing the two regimens. Many other cytokines were also substantially increased in coinfection (those significantly upregulated relative to both single infections are highlighted in Table EV1). To summarize, coinfection led to bacterial outgrowth which was closely associated with mortality. Coinfection induced severe disease and a fulminant immune response, comprised of massive recruitment of inflammatory monocytes, neutrophils and other immune cells, and strong upregulation of many proinflammatory cytokines including TNF-α, KC and MIP-2.

#### Absence of inflammatory monocytes and related cell populations increases survival and reduces lung damage and bacterial load in coinfection

**Figure 2:**
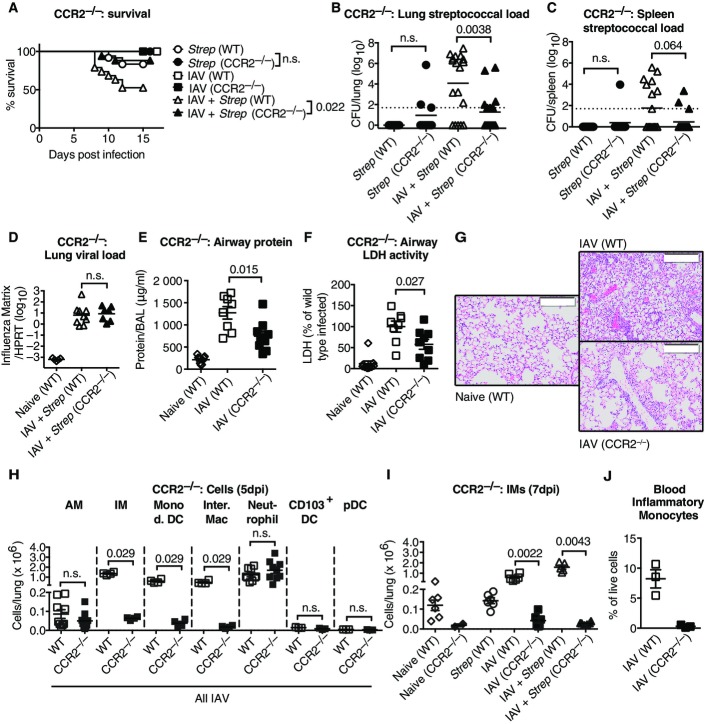
Mice deficient in inflammatory monocytes and related myeloid populations are resistant to coinfection and have reduced early lung damage A Mortality of CCR2^−/−^ and wild-type (WT) mice during low-dose coinfection (data shown are pooled from two independent experiments, *n* = 6–9).

Our results suggested that prior influenza infection allows bacterial outgrowth. We therefore sought to determine what factors contribute to disease severity or to protection in coinfection. To assess the role of inflammatory monocytes, we infected CCR2^−/−^ mice with a low-dose coinfection as described above. CCR2^−/−^ mice are deficient in peripheral inflammatory monocyte recruitment 22 since they lack the receptor for the chemoattractant MCP-1 (also known as CCL2). CCR2 deficiency ameliorated coinfection-induced mortality (Fig2A), and this improved survival coincided with reduced pneumococcal loads in the lung and a trend to reduced systemic spread (Fig2B and C). CCR2 deficiency did not alter susceptibility to the mild single *S. pneumoniae* and IAV infections used here (Fig2A) and did not affect viral load in coinfection (Fig2D).

B, C Pneumococcal load in the lung (B) and spleen (C) at 8 dpi during low-dose coinfection in CCR2^−/−^ and wild-type (WT) mice (data shown are pooled from two independent experiments; dotted line indicates detection limit, *n* = 4–9).

D Quantitative PCR for influenza matrix RNA in the lung during low-dose coinfection at 8 dpi in CCR2^−/−^ and wild-type (WT) mice (*n* = 5–9).

E, F Airway protein (E) and airway LDH activity relative to wild-type IAV-infected group mean (defined as 100%) (F) at 5 dpi in CCR2^−/−^ and wild-type (WT) mice (8 × 10^3^ TCID_50_) (data shown are pooled from three independent experiments, *n* = 2–3).

G H&E staining of lung tissue sections at 5 dpi in CCR2^−/−^ and wild-type (WT) mice (8 × 10^3^ TCID_50_). Scale bar indicates 200 μm (*n* = 2–3).

H Quantification of lung cells by flow cytometry at 5 dpi in CCR2^−/−^ and wild-type (WT) mice (8 × 10^3^ TCID_50_); AM = alveolar macrophages (Siglec F^+^CD11c^+^CD64^+^Ly6C^−^), IM = inflammatory monocytes (Siglec F^−^CD11b^+^MHCII^−^Ly6C^+^Ly6G^−^CD64^+^), Mono d. DC = monocyte-derived dendritic cells (Siglec F^−^CD11b^+^MHCII^+^CD11c^+^CD64^+^Ly6C^+^Ly6G^−^), Inter. Mac = interstitial macrophages (Siglec F^−^CD11b^+^MHCII^+^CD11c^−^CD64^+^Ly6C^+^), CD103^+^ DC = CD103^+^ dendritic cells (CD103^+^CD3^−^CD11c^+^CD24^+^Siglec F^−^CD11b^+^Ly6G^−^CD64^−^), pDC = plasmacytoid dendritic cells (PDCA-1^+^Ly6C^+^CD11c^int^CD11b^−^Siglec F^−^Ly6G^−^) (data shown are pooled from three independent experiments, *n* = 3–4).

I Quantification of lung inflammatory monocytes by flow cytometry at 7 dpi in CCR2^−/−^ and wild-type (WT) mice during high-dose coinfection (data shown are pooled from two independent experiments, *n* = 3).

J Quantification of blood inflammatory monocytes (as proportion of live cells) by flow cytometry at 5 dpi in CCR2^−/−^ and wild-type (WT) mice (8 × 10^3^ TCID_50_) (*n* = 3). Data information: Data are displayed as percentage survival (mortality), geometric means (viral and pneumococcal loads) or arithmetic means ± SEM (damage and cells). Significance was assessed by Mann–Whitney *U*-test (viral and pneumococcal loads, damage and cells) or log-rank (Mantel–Cox) test (mortality). n.s. = not significant.

We hypothesized that CCR2-mediated recruitment of inflammatory monocytes caused lung damage, which may promote bacterial colonization, explaining the protection seen in coinfected CCR2^−/−^ mice. To confirm this hypothesis, we assessed the extent of lung damage in wild-type and CCR2^−/−^ mice immediately prior to coinfection. CCR2^−/−^ mice had reduced epithelial leakage as measured by reduced total protein levels in the airway (Fig2E), decreased cell lysis as assessed by lactate dehydrogenase (LDH) activity (Fig2F), and less infiltrate and occlusion of airspaces as seen by histology (Fig2G) at 5 dpi, immediately prior to coinfection.

To identify the cell types that are involved in lung damage at day 5 and absent in infected CCR2^−/−^ mice, we used flow cytometry to extensively investigate immune cells recruited into the infected lungs of wild-type and CCR2^−/−^ mice on day 5 42, 43 (gating strategy shown in FigEV3). While we found no changes in lymphoid cell recruitment, a range of related myeloid cell subsets were dramatically reduced in CCR2^−/−^ lungs: inflammatory monocytes, monocyte-derived DCs and interstitial macrophages (Figs2H and EV4). Consistent with these results, we found the highest CCR2 expression on these cell types, with the exception of monocyte-derived DCs which likely have downregulated the receptor (FigEV4). However, the CCR2 dependence of recruitment of monocyte-derived DCs was in line with their monocyte origin. In contrast, alveolar macrophages and neutrophils expressed low CCR2 levels over unstained background levels, and their recruitment was CCR2 independent. Similarly, numbers of pDCs and conventional CD103^+^ DCs were unchanged in wild-type and CCR2^−/−^ mice (Fig2H). To exclude the possibility of CCR2-independent recruitment of inflammatory monocytes into coinfected lungs, we confirmed the near absence of these cells 2 days into the bacterial infection, at day 7 post-influenza infection (Fig2I). Another possibility was that monocytes accumulated in the blood to combat systemic bacterial spread, while staying outside the lung where they could contribute to damage. We found, however, no monocytes accumulating in the blood of CCR2^−/−^ mice at the time of coinfection (Fig2J).

**Figure:**
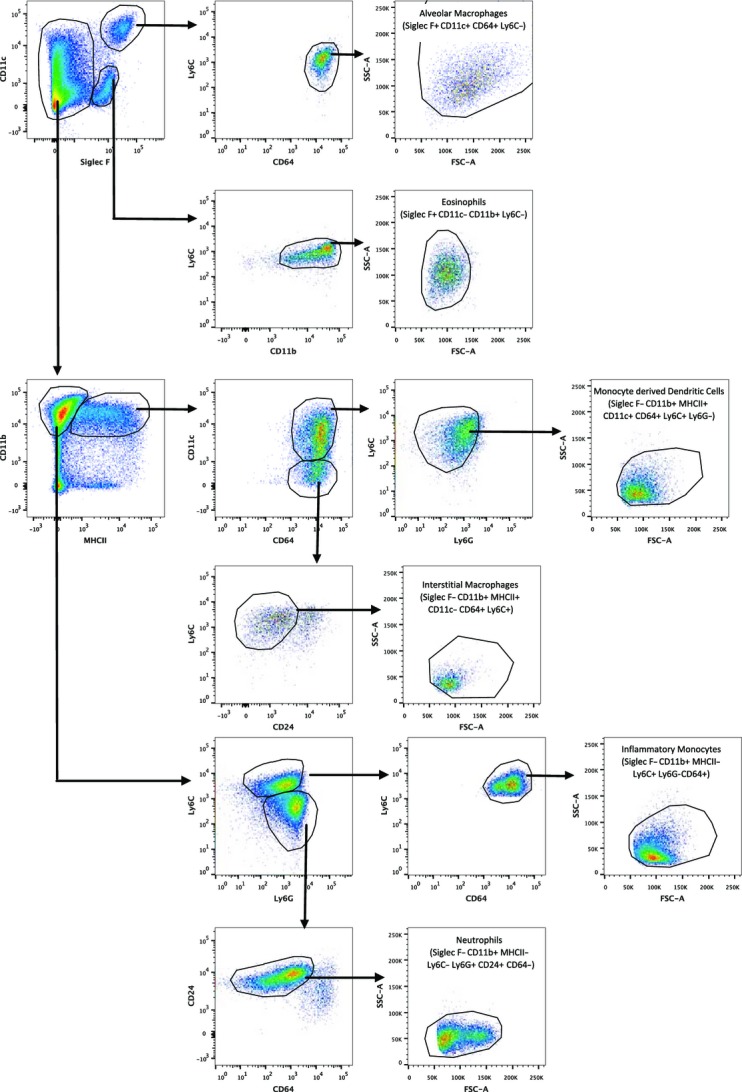
Myeloid flow cytometry gating strategy Flow cytometry gating strategy used for myeloid cells (neutrophils, alveolar macrophages, inflammatory monocytes and inflammatory monocyte-derived cells); pre-gated for live cells (Death Stain^−^ and exclusion of debris by size). Representative 5 dpi wild-type non-lavaged whole lung shown (8 × 10^3^ TCID_50_) (*n* = 4).

**Figure:**
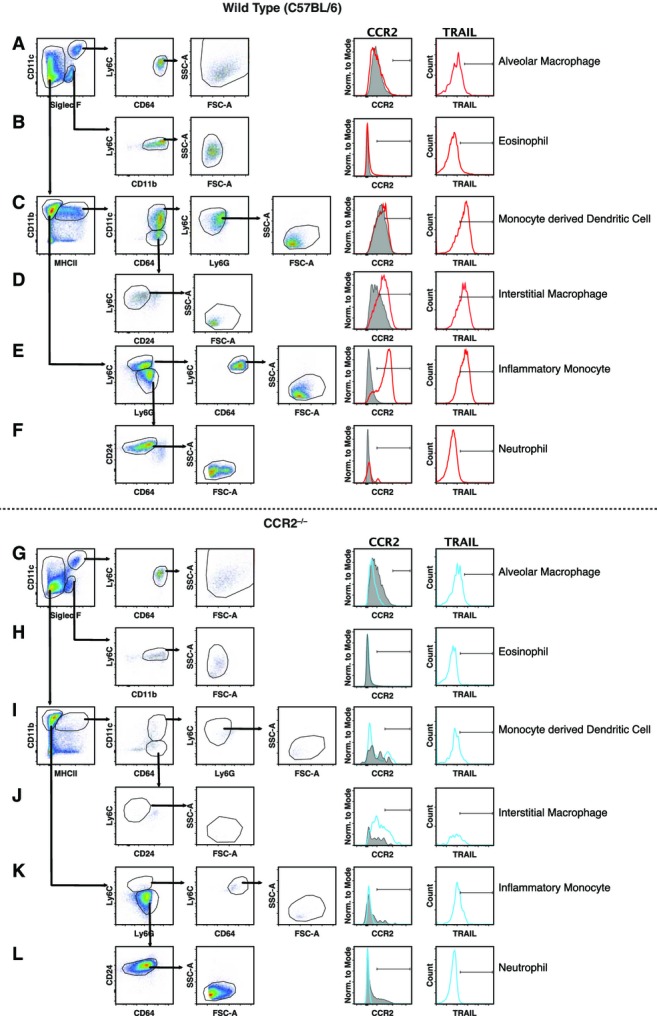
Monocytes and monocyte-related cells express TRAIL and are absent in CCR2^−/−^ mice A–F CCR2 and TRAIL expression in different myeloid cell populations as assessed by flow cytometry; pre-gated for live cells (Death Stain^−^ and exclusion of debris by size). Representative 5 dpi wild-type non-lavaged whole lung shown (8 × 10^3^ TCID_50_) (*n* = 4; note this lung is the same as shown in FigEV3).

G–L CCR2 and TRAIL expression in different myeloid cell populations as assessed by flow cytometry. Representative 5 dpi non-lavaged whole CCR2^−/−^ lung shown (8 × 10^3^ TCID_50_) (*n* = 4). Data information: Gating of each myeloid population is shown in the left panels, and histograms of CCR2 and TRAIL expression are shown on the right. Grey histogram represents unstained, and coloured histogram represents stained.

To conclude, CCR2 is required for the recruitment into the influenza-infected lung of three closely related myeloid cell types, namely inflammatory monocytes, monocyte-derived DCs and interstitial macrophages.

#### Inflammatory monocytes and related populations induce lung damage and increase susceptibility to bacterial colonization by a TRAIL-dependent mechanism

**Figure 3:**
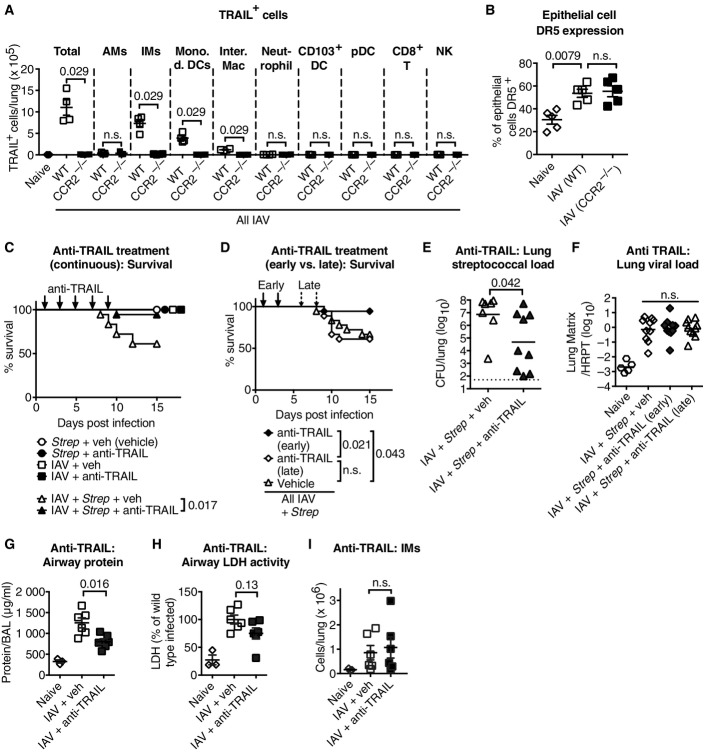
Blockade of TRAIL ameliorates coinfection A Quantification of TRAIL^+^ lung cells by flow cytometry at 5 dpi in CCR2^−/−^ and wild-type (WT) mice (8 × 10^3^ TCID_50_); abbreviations as in Fig2H (*n* = 4).

To determine the molecular mechanisms of inflammatory monocyte-mediated lung damage, we investigated the role of TRAIL (TNF-related apoptosis-inducing ligand), as TRAIL is a soluble or cell-surface molecule that is upregulated on inflammatory monocytes during severe influenza infection and can induce apoptosis of target cells expressing the TRAIL receptor DR5 25, 44. We first assessed the number of TRAIL-expressing cells recruited in CCR2^−/−^ mice immediately prior to coinfection (5 dpi) and found this number was substantially reduced compared to wild-type mice (Fig3A). This reduction was due to the absence of TRAIL-expressing myeloid cells including inflammatory monocytes, monocyte-derived DCs and interstitial macrophages, while the contribution of other cell types, including NK cells, pDCs and CD8 T cells, to the TRAIL^+^ cell population was negligible. Thus, there is a striking overlap of the myeloid cell types that express TRAIL (Figs3A and EV4) and those that depend on CCR2 to migrate into the infected lungs (Figs2H and EV4). Expression of the receptor DR5 on epithelial cells was unaffected by CCR2 deficiency (Fig3B).

B Quantification of the DR5^+^ proportion of lung epithelial cells (E-cadherin^+^Ep-Cam^+^) by flow cytometry at 5 dpi in CCR2^−/−^ and wild-type (WT) mice (8 × 10^3^ TCID_50_) (*n* = 5).

C Mortality during low-dose coinfection following treatment with anti-TRAIL or vehicle control (PBS) every 48 h from 1 to 9 dpi (in wild-type mice) (data shown are pooled from two independent experiments, *n* = 6–9).

D Mortality during low-dose coinfection following treatment with anti-TRAIL at 1 and 3 dpi (early), 6 and 8 dpi (late) or vehicle control (PBS) at 1, 3, 6 and 8 dpi (data shown are pooled from two independent experiments, *n* = 8–9).

E Lung pneumococcal load at 5 dpi + 16 h during high-dose coinfection, following treatment with anti-TRAIL at 1 and 3 dpi or vehicle (PBS) (dotted line indicates detection limit, *n* = 7–9).

F Quantitative PCR for influenza matrix RNA in the lung during low-dose coinfection at 8 dpi following treatment with anti-TRAIL at 1 and 3 dpi (early), 6 dpi (late) or vehicle control (PBS) at 1, 3 and 6 dpi (*n* = 5–10).

G, H Airway protein (G) and airway LDH activity relative to wild-type IAV-infected group mean (defined as 100%) (H) at 5 dpi (8 × 10^3^ TCID_50_) following treatment with anti-TRAIL or vehicle (PBS) at 1 and 3 dpi (*n* = 3–6).

I Quantification of lung inflammatory monocytes by flow cytometry at 5 dpi (8 × 10^3^ TCID_50_) following anti-TRAIL treatment at 1 and 3 dpi (*n* = 3–6). Data information: Data are displayed as mortality (survival), geometric mean (viral and bacterial loads) or arithmetic means ± SEM (damage and cells). Significance was assessed by log-rank (Mantel–Cox) test (mortality) or Mann–Whitney *U*-test (viral and bacterial loads, damage and cells). n.s. = not significant.

This suggests reduction in lung damage in CCR2^−/−^ mice may be due to reduced TRAIL ligand availability. To test this directly, we blocked TRAIL–DR5 interaction by use of a blocking antibody and found that anti-TRAIL treatment throughout viral and secondary bacterial infection (1–9 dpi) ameliorated the outcome of low-dose coinfection (Fig3C). To further investigate when the detrimental effect of TRAIL occurs, we treated mice with anti-TRAIL only during the viral phase (1 and 3 dpi, indicated as “early”) or only during the secondary bacterial infection phase (6 and 8 dpi, “late”). Only early treatment protected mice from coinfection (Fig3D). This strongly suggested that influenza-induced immune-mediated lung damage prior to coinfection, caused by TRAIL expression on myeloid inflammatory cells, allowed subsequent bacterial colonization upon secondary infection. To test this directly, we treated mice with anti-TRAIL during the viral phase and determined bacterial load at 16 h post-coinfection. As outlined previously (Fig EV1I), 16 h is the first time point where clear differences in bacterial load can be observed between singly and coinfected mice. At this early time point, anti-TRAIL treatment already reduced bacterial loads significantly (Fig3E), confirming that bacterial invasion depended on TRAIL-mediated damage. In contrast, neither early nor late TRAIL blockade affected viral load in coinfection (Fig3F).

Treatment with anti-TRAIL was protective since it reduced damage at the point of coinfection as assessed by airway protein (Fig3G), and there is a trend to reduced LDH activity (Fig3H). We therefore conclude that TRAIL-expressing myeloid cells comprising inflammatory monocytes and monocyte-derived cells cause epithelial cell death during mild influenza infection, leading to increased lung damage, which allows bacterial colonization upon coinfection.

It is possible, as inflammatory monocytes express TRAIL on their surface, that treatment with an anti-TRAIL antibody could lead to depletion of this population. This may account for the protective effect seen in anti-TRAIL-treated mice. However, treatment with anti-TRAIL did not change inflammatory monocyte numbers at the point of coinfection (Fig3I). Therefore, the protective effect of anti-TRAIL treatment can be considered a genuine effect of TRAIL blockade.

#### Neutrophils are essential for survival and contribute to bacterial control in coinfection

**Figure 4:**
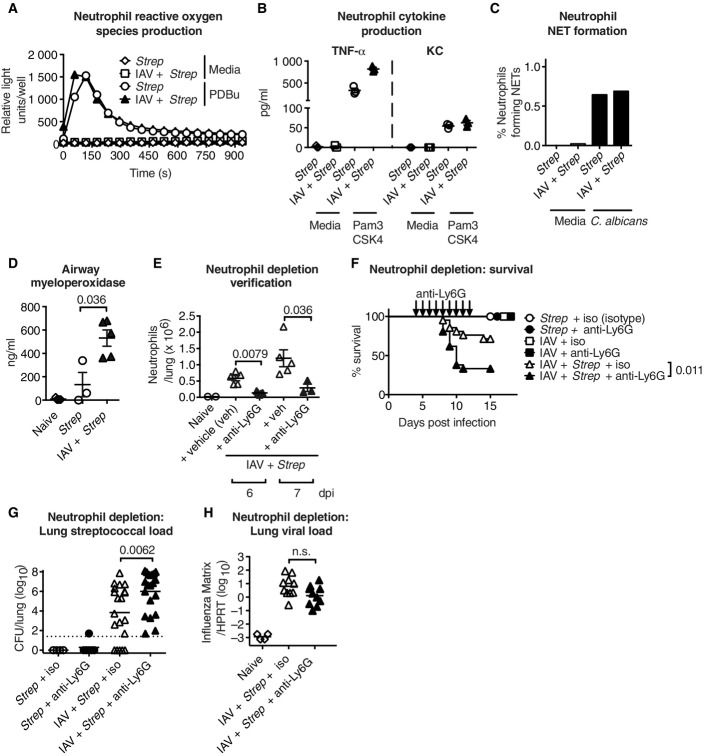
Neutrophils from coinfected mice are functional and contribute to survival and bacterial control ROS production was assessed by luminol assay of PDBu (50 nM)-stimulated lung neutrophils purified by MACS from high-dose-coinfected or *S. pneumoniae-*infected mice at 6 dpi (neutrophils from nine mice pooled into three replicates/group).

Having determined a novel upstream immune mechanism promoting bacterial colonization in coinfection, we then investigated the effects of components of the downstream immune response to coinfection. The massive recruitment of neutrophils and upregulation of TNF-α upon coinfection raised the question of whether these responses are on balance protective or damaging. While neutrophils have been proposed to protect in single bacterial infections, it has also been suggested that neutrophil function may be impaired in coinfection 33. To test this, we purified neutrophils from *S. pneumoniae*-infected and *S. pneumoniae*-coinfected mouse lungs and tested their functionality. Neutrophils showed no difference in ROS production, ability to produce cytokines or neutrophil extracellular trap 7 formation (Fig4A–C) in response to different stimuli *in vitro*. We also found strongly increased myeloperoxidase (MPO) activity in BAL fluid from coinfected compared to singly infected mice (Fig4D), consistent with strong recruitment of functional neutrophils in coinfection. Histology indicates that MPO-positive neutrophils with characteristic polymorphic nuclei contributed to bacterial elimination by phagocytosis (Fig EV5A), and we confirmed *in vivo* that even in coinfection, NET formation was not induced by *S. pneumoniae* (FigEV5B), consistent with our *in vitro* results showing that neutrophils from coinfected lungs were able to form NETs, but it takes a stimulus different from *S. pneumoniae* to induce this (Fig4C). We conclude from these *in vitro* and *in vivo* data that neutrophils are not functionally impaired in coinfection.

ELISA quantification of TNF-α and KC produced by Pam3CSK4 (1 μg/ml)-stimulated neutrophils purified by MACS from high-dose-coinfected or *S. pneumoniae-*infected mice at 6 dpi (neutrophils from three mice/group).

Percentage of NET-forming cells was assessed by microscopy of *C. albicans-*stimulated (5 × 10^5^ CFU) neutrophils purified by MACS and Percoll gradient from high-dose-coinfected or *S. pneumoniae-*infected mice at 6 dpi (neutrophils from three mice pooled/group).

ELISA quantification of airway myeloperoxidase at 6 dpi during high-dose coinfection (*n* = 3–5).

Quantification of lung neutrophils (without staining for Ly6G—CD11b^+^SSC^>low^CD11c^−^MHCII^−^Ly6C^low^F4/80^−^) by flow cytometry at 6 and 7 dpi during low-dose coinfection following treatment with anti-Ly6G or vehicle control every 24 h from 4 dpi (*n* = 3–5).

Mortality during low-dose coinfection following treatment with anti-Ly6G or isotype control every 24 h from 4 to 12 dpi (data shown are pooled from two independent experiments, *n* = 9).

Lung pneumococcal load at 8 dpi during low-dose coinfection following treatment with anti-Ly6G or isotype control (data shown are pooled from two independent experiments; dotted line indicates detection limit, *n* = 5–10).

Quantitative PCR for influenza matrix RNA in the lung during low-dose coinfection at 8 dpi following treatment with anti-Ly6G or isotype control every 24 h from 4 to 7 dpi (*n* = 4–10). Data information: Data are displayed as arithmetic means ± SEM (ROS, cytokine production, myeloperoxidase and neutrophil numbers), percentage of neutrophils (NET formation), percentage survival (mortality) or geometric means (viral and pneumococcal loads). Significance was assessed by Mann–Whitney *U*-test (myeloperoxidase, neutrophil numbers, viral and pneumococcal loads) or log-rank (Mantel–Cox) test (mortality). n.s. = not significant.

**Figure:**
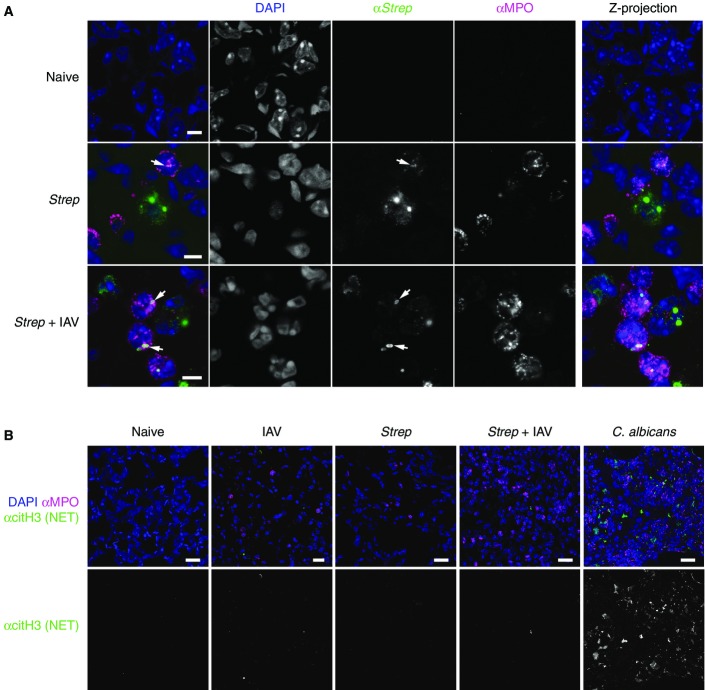
Neutrophils phagocytose streptococci but do not produce NETs during coinfection Confocal microscopy of lung tissue sections at 8 dpi during high-dose coinfection stained for cell nuclei (DAPI), streptococci (α*Strep*) and neutrophils (αMPO). Black text indicates infection condition; coloured text indicates staining. Right column shows the Z-projection of 10 individual focal planes; other columns show a single plane. Arrows indicate bacteria phagocytosed by neutrophils. Scale bars indicate 5 μm (*n* = 3).

Confocal microscopy of lung tissue sections at 8 dpi during high-dose coinfection (or during *C. albicans* infection as positive control) stained for cell nuclei (DAPI), neutrophils (αMPO) or the NET constituent citrullinated histone H3 (αcitH3). Black text indicates infection condition; coloured text indicates staining. Scale bars indicate 20 μm (*n* = 3).

Having established abundant recruitment of fully functional neutrophils to the lung, we tested directly the damaging or protective effect of neutrophils. We depleted neutrophils during low-dose coinfection immediately prior to and during secondary bacterial infection (4–10 dpi) using a monoclonal antibody against Ly6G, a marker specifically expressed on mouse neutrophils 45 and confirmed depletion by a flow cytometric staining not employing anti-Ly6G (Fig4E). Depletion of neutrophils increased mortality during coinfection (Fig4F), and consistent with the strong association between mortality and bacterial outgrowth (Fig1F and G), pneumococcal load was increased 140-fold in the coinfected lung (Fig4G). Notably, neutrophil depletion did not affect survival or bacterial load in single *S. pneumoniae* infection and did not affect viral load in coinfection (Fig4H). Therefore, functional neutrophils recruited to the lung play an overall protective role in coinfection by helping control bacterial load.

#### TNF-α is essential for survival and bacterial control in coinfection

**Figure 5:**
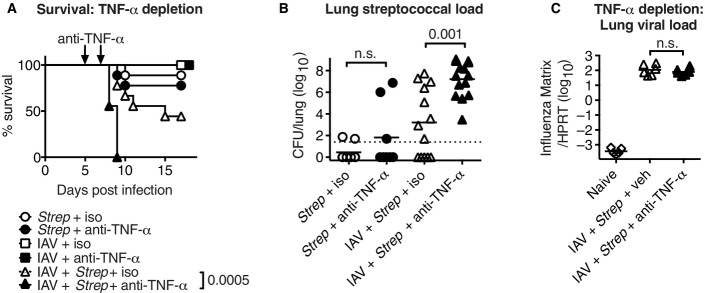
TNF-α contributes to survival and bacterial control in coinfected mice Mortality during low-dose coinfection following treatment with anti-TNF-α or isotype control at 5 and 7 dpi (*n* = 6–9).

In addition to the neutrophil response, we also observed strong induction of TNF-α in coinfected lungs as compared to the single infections. TNF-α is a proinflammatory cytokine which is protective during virulent *S. pneumoniae* infection 37. To test whether TNF-α played a role in coinfection, we treated mice in a low-dose coinfection regimen with anti-TNF-α immediately prior to and during secondary bacterial infection (5 and 7 dpi). Blockade of TNF-α signalling increased mortality (Fig5A), which associated with higher pneumococcal load in the lung (Fig5B). Importantly, anti-TNF-α treatment did not affect survival or bacterial load in single *S. pneumoniae* infection (Fig5B) or viral load in coinfection (Fig5C). We conclude that TNF-α is required for bacterial control in coinfection and is therefore protective.

Pneumococcal load in the lung at 8 dpi during low-dose coinfection following anti-TNF-α or isotype control treatment (data shown are pooled from two independent experiments; dotted line indicates detection limit, *n* = 3–9).

Quantitative PCR for influenza matrix RNA in the lung during low dose at 7 dpi following treatment with anti-TNF-α or vehicle control at 5 and 7 dpi (*n* = 5–6). Data information: Data are displayed as percentage survival (mortality) or geometric means (viral and pneumococcal loads). Significance was assessed by Mann–Whitney *U*-test (viral and pneumococcal loads) or log-rank (Mantel–Cox) test (mortality).

### Discussion

**Figure 6:**
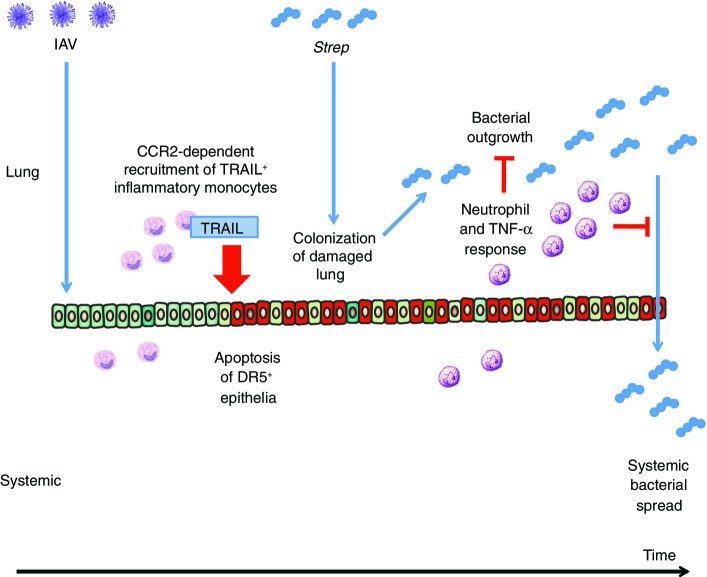
Summary of coinfection pathogenesis Influenza infection leads CCR2-dependent recruitment of monocytes and other monocyte-related immune cell populations into the lung. These cells express TRAIL and induce epithelial apoptosis causing lung damage, thus facilitating bacterial invasion due to breakdown of barrier function. The subsequent bacterial outgrowth drives strong TNF-α and neutrophil responses which are on balance protective as they contribute to the (limited) control of bacteria.

Here we demonstrate, in a coinfection model of moderate severity, a new mechanism of susceptibility—immune-mediated damage allowing bacterial colonization. We also use this model to show conclusively that the massive amounts of neutrophils and TNF-α induced during coinfection are on balance protective, not deleterious (Fig6).

In this study, moderate influenza infection renders mice susceptible to an otherwise mild *S. pneumoniae* coinfection, a situation that mirrors the coinfection events in regular influenza seasons and in the 2009 pandemic 6, 7, 8. In these situations, influenza was caused by virus strains that are not as highly pathogenic as some avian and the 1918 pandemic influenza strains. Coinfection is characterized by a loss of bacterial control in the lung and systemic spread of bacteria. Coinfected CCR2^−/−^ mice lacking inflammatory monocytes and related populations have reduced lung damage prior to secondary infection, control bacterial outgrowth and thus are protected. TRAIL is mainly expressed on those monocyte-related populations whose recruitment required CCR2, and anti-TRAIL-treated mice are also resistant to coinfection. We find that anti-TRAIL treatment is protective when given during influenza infection, but not when given during subsequent bacterial coinfection. Early anti-TRAIL treatment reduces the ability of *S. pneumoniae* to invade the influenza-infected lung, leading to lower bacterial loads. We therefore identify upstream mechanisms in the immune response to influenza that facilitate secondary bacterial infection. We also show that bacterial outgrowth induces a strong downstream immune response that contributes to protection: since depletion of neutrophils or TNF-α exacerbates disease, the net effect of these responses is protective. Together, our results indicate that influenza-induced TRAIL-expressing inflammatory monocytes, monocyte-derived DCs and interstitial macrophages cause lung damage, which allows bacterial colonization upon secondary infection. This leads to a strong immune response comprising neutrophils and TNF-α that contribute to bacterial control but are, however, frequently insufficient to eliminate bacteria (Fig6).

Our model represents a substantial advance in determining the causes of death in coinfection. We address the multifactorial nature of coinfection by separating upstream causes of bacterial colonization from downstream responses to bacterial outgrowth. Furthermore, our 50% mortality regimen enables us to use interventions to determine both protective and pathogenic aspects of the immune response. Moreover, we employ a model of moderate influenza infection where blocking TRAIL or monocytes does not alter susceptibility to the virus. Even during such moderate viral infection, TRAIL-expressing inflammatory monocytes and related myeloid populations induce sufficient epithelial damage to allow subsequent bacterial colonization. Bacterial outgrowth then prompts a strong immune response, components of which we show are protective in coinfection.

In our coinfection model of 50% mortality, we find a tight correlation between individual mice reaching clinical endpoint and having high bacterial load. Consistent with these results, most IAV–*S. pneumoniae* coinfection studies show 100% mortality associated with a loss of bacterial control across the whole treatment group. However, in some other systems, such as a *L. pneumophila* model, tissue damage in the absence of massive bacterial outgrowth is the cause of mortality 46. In our system, secondary bacterial infection does not affect viral clearance, although in a model with a more virulent influenza strain, coinfection delays viral clearance 12. The strong association of mortality with bacterial outgrowth, and the inability of heat-killed bacteria or TLR2 agonist to replace live bacteria in inducing mortality, strongly suggests bacterial outgrowth and systemic spread—but not exaggerated immune responses—are the prime drivers of mortality. Furthermore, a recent study showing that immunopathology-reducing dexamethasone treatment is only effective once bacterial outgrowth has been controlled by antibiotics supports this view 35.

Inflammatory monocytes may comprise or develop into Tip-DCs/monocyte-derived DCs or exudate/interstitial macrophages. In our mild to moderate infection conditions, it appears that all TRAIL-expressing cells found in the infected lung belong to this group of closely related myeloid populations, and that these subsets all require CCR2 for their recruitment into the infected lung, similar to what was described for recruitment into other organs 42. Since CCR2 expression on monocyte-derived DCs is not above background, but their recruitment is CCR2 dependent, it is likely that these cells lose receptor expression in the lung upon differentiation from monocytes.

Tip-DCs and exudate macrophages have been shown to cause damage in severe influenza 26, but are also required for a full CD8^+^ T-cell response in lethal infection 27. Expression of TRAIL—a cell surface or soluble ligand that activates apoptotic pathways via DR5—on inflammatory monocytes causes airway epithelial cell apoptosis during severe influenza 25, 30, while TRAIL was shown to contribute to protection in other studies of influenza infection 31, 47. TRAIL^−/−^ mice have been reported to be more susceptible to a severe single *S. pneumoniae* infection 29. Here we show that CCR2^−/−^ mice—which do not recruit inflammatory monocytes to the lung—and anti-TRAIL-treated mice are resistant to coinfection. In contrast to the above studies using severe influenza models, the absence of inflammatory monocytes and related populations or the blockade of TRAIL has no effect on susceptibility to the mild to moderate single infections used here. Our results differ from those in a similar study 25 which found TRAIL upregulation on airway macrophages in severe but not in mild influenza infection. In contrast to our study, Herold *et al* focused on TRAIL-expressing Gr-1^int^ cells which would exclude some of the CCR2-dependent TRAIL-expressing populations assessed here (Ly6C^high^ cells). Other differences including virus dose and route and volume of inoculum may contribute to divergent results. In conclusion, in mild influenza infection, we find an almost complete overlap between CCR2 dependency and TRAIL expression among monocyte-derived populations. In addition, we identify immunopathological mechanisms that are too subtle to change the course of mild to moderate single infections as crucial determinants of severe outcome in coinfection. In more severe influenza models, it has been shown that pharmacological CCR2 blockade can reduce disease severity only when given 1 day prior to influenza exposure 48. In the light of our results, CCR2 blockade even after influenza exposure may have a beneficial effect as it may reduce the risk of subsequent bacterial infection.

Expression of TRAIL during influenza could also contribute to coinfection susceptibility through other mechanisms. Expression of DR5 is not limited to epithelial cells, and therefore, TRAIL could induce apoptosis of immune cells that may be required for bacterial control, such as alveolar macrophages 29, and depletion of alveolar macrophages by influenza has been proposed as disease promoting in coinfection 18. However, although at the point of secondary infection (5 dpi), we observe a trend to reduced alveolar macrophage numbers, their numbers are similar in resistant CCR2^−/−^ and susceptible wild-type mice. Apoptotic cells expressing CD200 can reduce the innate immune response to secondary bacterial infection 10. TRAIL-mediated apoptosis may exacerbate this and may therefore represent a contributing factor. Notably, as TRAIL expression during influenza infection is type I IFN dependent 44, the mechanism described here likely contributes to the reduced susceptibility of IFNαβR^−/−^ mice to coinfection previously reported 14.

Many studies attribute coinfection susceptibility to influenza-induced immune impairment via various mechanisms. In our model, we do not observe immune impairment in the aspects profiled; bacterial outgrowth induces a strong inflammatory response characterized by neutrophils and proinflammatory cytokines such as TNF-α. In our model of moderate coinfection mortality that allows us to monitor both beneficial and detrimental effects of experimental intervention, we show that specific depletion of neutrophils or depletion of TNF-α exacerbates bacterial outgrowth. In contrast, depletion of neutrophils and TNF-α in the single mild *S. pneumoniae* infection had little to no effect. Thus, we are able to identify protective factors in coinfection, such as neutrophils and TNF-α, which are not required for protection from the single mild *S. pneumoniae* infection employed here.

Previous studies have assessed the role of neutrophils in influenza–*S. pneumoniae* coinfection. IFNαβR^−/−^ mice have greater KC and MIP2 production and neutrophil recruitment in coinfection, correlating with increased survival 14. Treatment with the broad-range mAb anti-Gr-1 (RB6), which depletes neutrophils as well as plasmacytoid dendritic cells, some inflammatory monocytes and lymphocytes 45, increased bacterial outgrowth at 24 h post-secondary infection 13. In another study, anti-Gr-1 treatment increased bacterial load in a coinfection given at 3 days, but not at 6 days post-influenza 33. This study proposes that depletion has no effect at 6 days after influenza infection, as neutrophils are already functionally impaired, and therefore not protective. As anti-Gr-1 depletion is poorly specific, it is possible that a beneficial, damage-reducing effect of monocyte depletion and a negative effect of neutrophil depletion on bacterial control overlay each other, making these results difficult to interpret. Here we separately block monocyte recruitment by the use of CCR2^−/−^ mice and specifically deplete neutrophils by an antibody specific for these cells and are therefore able to distinguish the divergent effects of these two cell subsets.

Furthermore, the above infection models often use more virulent IAV and *S. pneumoniae* strains than those in our experiments, which may represent the clinical observations in coinfection associated with highly pathogenic viruses as in the 1918 pandemic. Milder infection may also explain why we do not observe neutrophil impairment. In the above model 33, lung neutrophils are elicited for functional tests using LPS aerosolization, which may affect their function. Another study 35 depletes neutrophils in conditions where all mice reach endpoint and show high bacterial loads; therefore, unlike in our model, there is no further margin to exacerbate coinfection by neutrophil depletion. In influenza–*Staphylococcus aureus* coinfection, neutrophil depletion strongly increases susceptibility in some studies 49, while in others, it has less dramatic effects than that shown here 50, which may reflect the ability of some *S. aureus* strains to block neutrophil function 51. To summarize, through specific neutrophil depletion, we demonstrate that the net effect of neutrophils is protective in influenza–*S. pneumoniae* coinfection of moderate severity, representative of the clinical situation in seasonal influenza and the 2009 pandemic.

The role of TNF-α in coinfection is not well established; however, previous studies have reported a rise in TNF-α levels during coinfection 11, 36. One study shows that TNF-α and IL-1β production is somewhat reduced following coinfection 13; we did not observe this. A lethal IAV–*H. influenzae* coinfection model reported no effect of TNFR1 deficiency 52, again in a high mortality setting where there was no margin to see exacerbation of disease upon intervention. Thus, our model is the first to directly demonstrate that on balance, the strong TNF-α response to influenza–*S. pneumoniae* coinfection is protective, rather than a driver of immunopathology. This is in agreement with a study in influenza–*S. aureus* coinfection where NK-cell-derived TNF-α was shown to contribute to bacterial control 53.

In conclusion, through detailed profiling of influenza–*S. pneumoniae* coinfection and multiple interventions, we propose a novel mechanism of immune-mediated damage as determinant of disease severity in coinfection. We show that influenza-induced TRAIL-expressing inflammatory monocytes and related populations cause lung damage that allows bacterial colonization of the lung and subsequent bacterial outgrowth. Outgrowth induces a strong immune response including neutrophil recruitment and TNF-α induction, which we show contribute to bacterial control and are protective in coinfection. The identification of disease-promoting and protective components of the immune response to coinfection will inform future treatment strategies to combat this important public health burden.

## Materials and Methods

### Mice and infections

All experiments used 6- to 10-week-old C57BL/6 or CCR2^−/−^ (C57BL/6) (Jackson Laboratory) mice bred at the MRC National Institute for Medical Research (NIMR) under specific pathogen-free conditions. Influenza A virus strain X31 (H3N2) (a reassortment virus with A/PR/8/34 backbone) was grown in day 10 embryonated chicken eggs, stored at −80° and titrated on MDCK cells (ATCC) to establish 50% tissue culture infective dose (TCID_50_), according to the Spearman-Karber method. *Streptococcus pneumoniae* D39 (a kind gift from Dr. M. Coles, University of York) was stored at −80° on cryopreservative beads (Technical Services Consultants) and grown in brain–heart infusion broth under microaerophilic conditions at 37° for 16 h to autolytic phase, then subcultured and grown to an optical density of 0.4, centrifuged and resuspended in PBS immediately prior to infection. Where described as “heat killed,” the bacteria were incubated at 80° for 10 min prior to infection. Mice were infected under light isoflurane-induced anaesthesia intranasally (i.n.) with a 30-μl volume.

### Clinical scoring and endpoints

Mice were deemed to have reached endpoint at 75% of starting weight or at a moderate severity clinical score of 5 or greater. Clinical scores were determined by (1 point each) piloerection, hunched posture, laboured breathing, decreased movement, movement only on provocation, absence of movement on provocation, hypothermia and partially closed eyes. No blinding was performed when assessing clinical scores.

### Ethics statement

All protocols for breeding and experiments with animals were approved by the Home Office, UK, under the Animals (Scientific Procedures) Act 1986 and project licence 70/7643.

### Mouse treatments

All antibody treatments were given intraperitoneally (i.p.) in a 200-μl volume. A total of 150 μg anti-Ly6G (clone 1A8) or isotype control (2A3) (BioXCell) was given every 24 h from 4 to 12 dpi. A total of 500 μg anti-TNF-α (XT3.11) or isotype control (HRPN) (BioXCell) was given on 5 and 7 dpi. A total of 150 μg anti-TRAIL (Cambridge Bioscience) or vehicle (PBS) was given either (continuous treatment) every 48 h from 1 to 9 dpi, (early treatment) at 1 and 3 dpi or (late treatment) at 6 and 8 dpi.

### Bacterial loads

Pneumococcal loads were determined by homogenization of lung or spleen tissue in PBS through a 70-μm filter prior to storage at −80°. Serial dilutions of single–cell suspensions or bronchoalveolar lavage (BAL) fluid were performed on brain–heart infusion agar plates supplemented with defibrinated horse blood and the number of colony-forming units (CFUs) counted.

### Viral and bacterial RNA quantification

RNA was extracted from lung using TRI reagent (Ambion) as per the manufacturer’s instructions. Four hundred nanograms of total RNA was reverse-transcribed using the ThermoScript RT-PCR System kit (Invitrogen) as per the manufacturer’s instructions. The cDNA served as a template for quantitative PCR using TaqMan Gene Expression Assays (Applied Biosystems), universal PCR Master Mix (Applied Biosystems) and the ABI-PRISM 7900 sequence detection system (Applied Biosystems). IAV Matrix M1 RNA and *S. pneumoniae* 16S rRNA were quantified relative to the housekeeping gene (Hprt1) as previously described 54, 55. Primers for influenza matrix M1 gene were as follows:

forward: 5′-AAGACCAATCCTGTC ACCTCTGA-3′;
reverse: 5′-CAAAGCGTCTACGCTGCAGTCC-3′;
and probe: 5′-TTTGTGTTCACGCTCACCGT-3′.

Primers for *Strep* 16S rRNA were as follows:

forward: 5′-GGTGACGGC AAGCTAATCTCTT-3′;
reverse: 5′-AGGCGAGTTGCAGCCTACAA-3′;
and probe: 5′-AAGCCAGTCTCAGTTCG-3′.

### Histology

Whole lungs were perfused with 10% neutral-buffered formalin (NBF) *in situ*. Tissue was then fixed overnight in 10% NBF, transferred into ethanol until embedded in paraffin and sectioned. Each lung specimen was stained with haematoxylin and eosin (H&E). Imaging of slides was performed on a VS120 slide scanner (Olympus) with a VC50 camera, a UPLSAPO lens, at a magnification of 20× and a numerical aperture of 0.75. Images were analysed using OlyVia Image Viewer 2.6 (Olympus).

### Flow cytometry

Leucocytes from the lung and blood were enumerated using flow cytometry. Lungs were excised from mice, digested with 20 μg/ml Liberase TL 21 and 50 μg/ml DNase 1 (Sigma) and homogenized using gentleMACS (Miltenyi), as per the manufacturer’s instructions. For analysis of epithelial cells, gentleMACS homogenization was not performed. Lungs were then passed through a 70-μm cell strainer and washed with PBS. Red blood cells were lysed for 5 min with ammonium chloride, and cells were seeded into a 96-well U-bottom plates. Blood was removed from mice by cardiac puncture and collected into cold heparin. Red blood cells were lysed using BD Red Blood Cell Lysis solution (as per the manufacturer’s instructions), and cells were seeded onto 96-well U-bottom plates. Cells were preincubated with anti-FcγRIII/II (Fc block) in PBS followed by staining for cell death with LIVE/DEAD® AmCyan Fixable Dead Cell Stain (Life Technologies), prior to 30-min incubation with one or more of the following fluorochrome-labelled antibodies (Biolegend unless otherwise specified): FITC-conjugated anti-Ly6G; APCCy7-conjugated Ly6G; AF700-conjugated Ly6G; PerCpCy5.5-conjugated anti-Ly6C; FITC-conjugated anti-Ly6C; PECy7-conjugated anti-CD11b; APC-conjugated anti-CD11b; BV711-conjugated anti-CD11b; BV605-conjugated anti-CD11c; V450-conjugated anti-CD11c (BD Biosciences); BV421-conjugated anti-CD103; APCCy7-conjugated anti-CD3; AF700-conjugated CD3; PECy7-conjugated anti-CD64; FITC-conjugated anti-CD24; PE-conjugated anti-F4/80; FITC-conjugated MHCII; APC-conjugated anti-CCR2 (R and D systems); PE-conjugated anti-TRAIL; PE-conjugated anti-DR5; APC-conjugated CD45; Pacific Blue-conjugated CD45.2; APCCy7-conjugated anti-EpCam; V450-conjugated anti-CD4; BV605-conjugated anti-CD4; PerCpCy5.5-conjugated anti-NKp46; FITC-conjugated anti-NKp46; PECy7-conjugated anti-NK1.1; BV650-conjugated anti-NK1.1; FITC-conjugated anti-E-cadherin; BV650-conjugated anti-PDCA-1; BV786-conjugated anti-CD8; PE-Texas Red-conjugated anti-CD8 (Life Technologies); and biotin-conjugated anti-Siglec F (Miltenyi Biotec). Cells were then washed and stained with PE/Dazzle 594 streptavidin (Biolegend) and incubated for further 20 min on ice. Cells were washed again with PBS before fixation in 4% formaldehyde and were then assessed using a LSR II Fortessa or Fortessa X20 (Becton Dickinson). Analysis was performed on FlowJo (Treestar). Cell counts were performed on a Brightline hemacytometer (Hausser Scientific) with trypan blue exclusion.

### Airway protein quantification

Bronchoalveolar lavage fluid was recovered and centrifuged at 1,300 rpm for 5 min at 4°C and supernatant collected. Concentrations of cytokines were assessed by Milliplex Map Kit (Millipore) as per the manufacturer’s instructions and read on a Luminex 100 (Bio-Rad). Concentrations of MPO were quantified by ELISA (R & D systems) according to the manufacturer’s protocol.

### Quantification of lung damage

Lung damage was assessed in BAL fluid. LDH activity was assessed using the enzymatic detection step of the CytoTox 96 Non-Radioactive Cytotoxicity Assay (Promega) according to the manufacturer’s instructions. Protein levels were quantified by Pierce BCA protein assay (Thermo Scientific) as per the manufacturer’s instructions. All plates were read on a Safire II plate reader (Tecan).

### Neutrophil purification

Neutrophils were purified from whole mouse lung by dissection followed by 20 min of digestion with collagenase D 21 (0.5 mg/ml), dispase II 21 (2 mg/ml) and DNase 1 (Sigma) (3.5 μg/ml) with EDTA (Sigma) (10 μM) added for the final 5 min. Digested lung was mashed through a 70-μm filter, and neutrophils were separated from the single-cell suspension by positive selection on a MACS column using anti-Ly6G-biotin and anti-biotin microbeads (Miltenyi Biotec), as per the manufacturer’s instructions. Where specified, remaining dead cells were removed on a 40/85% Percoll gradient by retaining the fraction at the interface.

### Reactive oxygen species

A total of 5 × 10^4^ neutrophils were seeded on a white 96-well flat-bottomed plate in calcium- and magnesium-positive media and rested at 37° for 1 h. Luminol (Sigma) (50 μM) and horseradish peroxidase (Sigma) (1.2 U/ml) were added followed by stimulation with PDBu (Sigma) (50 nM) or media. Luminescence was immediately read on a Safire II plate reader (Tecan).

### NET formation (*in vitro*)

A total of 5 × 10^4^ neutrophils were seeded onto a 24-well flat-bottomed transparent plate in calcium- and magnesium-positive media supplemented with 3% mouse plasma and rested at 37° for 1 h, followed by stimulation for 2 h with 5 × 10^5^ CFU *C. albicans* (clinical isolate SC 5314) or media. After 2 h of incubation at 37°, NET formation was visualized by addition of the DNA stain Sytox (Life Technologies) (8.3 μM). Images were taken and analysed on a DM IRB (Leica) microscope with an Orca-ER Digital Camera C4742 80 (Hamamatsu) and an N PLAN PH1 lens (Leica), at a magnification of 10× and a numerical aperture of 0.25. Image acquisition software was Micromanager 1.4 and processing was performed using ImageJ 1.64. NETs were defined as Sytox^+^ areas > 2,000 μm^2^.

### Neutrophil culture

A total of 1 × 10^5^ neutrophils were seeded onto a 96-well flat-bottomed plate in complete media and rested for 1 h, followed by stimulation with Pam3CSK4 (Enzo Life Sciences) (1 μg/ml) or media. Culture supernatants were taken after 5 h following cell adhesion to the culture plate. Concentrations of TNF-α (e-Bioscience) and KC (R & D systems) were quantified by ELISA according to the manufacturer’s protocol.

### Neutrophil phagocytosis and NET formation (*in vivo*)

Whole lungs were perfused with 10% neutral-buffered formalin (NBF) *in situ*. Tissue was then fixed overnight in 10% NBF, embedded in paraffin and sectioned. Lung sections were treated with a standard antigen retrieval and immunofluorescence staining protocol: DAPI (Life Technologies), anti-myeloperoxidase (anti-MPO) (R&D Systems), anti-*Streptococcus pneumoniae* type 2 rabbit polyclonal serum (anti-*Strep*, Abcam), donkey anti-goat IgG (H+L) secondary antibody Alexa Fluor 568 conjugate (Life Technologies) and donkey anti-rabbit IgG (H+L) secondary antibody Alexa Fluor 488 conjugate (Life Technologies); or DAPI, anti-MPO, anti-citrullinated histone 3 (anti-citH3, citrulline R2 + R8 + R17) (Abcam) and the same secondary antibody as above. Stained tissues were mounted and examined with confocal microscopy. Image analysis was performed using ImageJ 1.64.

### Statistics

All statistical comparisons were performed using Prism 6 (GraphPad). A *P*-value of < 0.05 was considered significant. n.s. = not significant.

## Supporting Information

**Expanded View Figures PDF**

**Table EV1**

**Review Process File**
