# Supplementary material for: TRAIL+ monocytes and monocyte-related cells cause lung damage and thereby increase susceptibility to influenza–Streptococcus pneumoniae coinfection
Source: EMBO Rep. 2015 Aug 18;16(9):1203–18. doi: 10.15252/embr.201540473 (PMC4576987; doi:10.15252/embr.201540473)
Supplement: Supplementary file 1 [file embr0016-1203-sd1.pdf]

Expanded View Figures

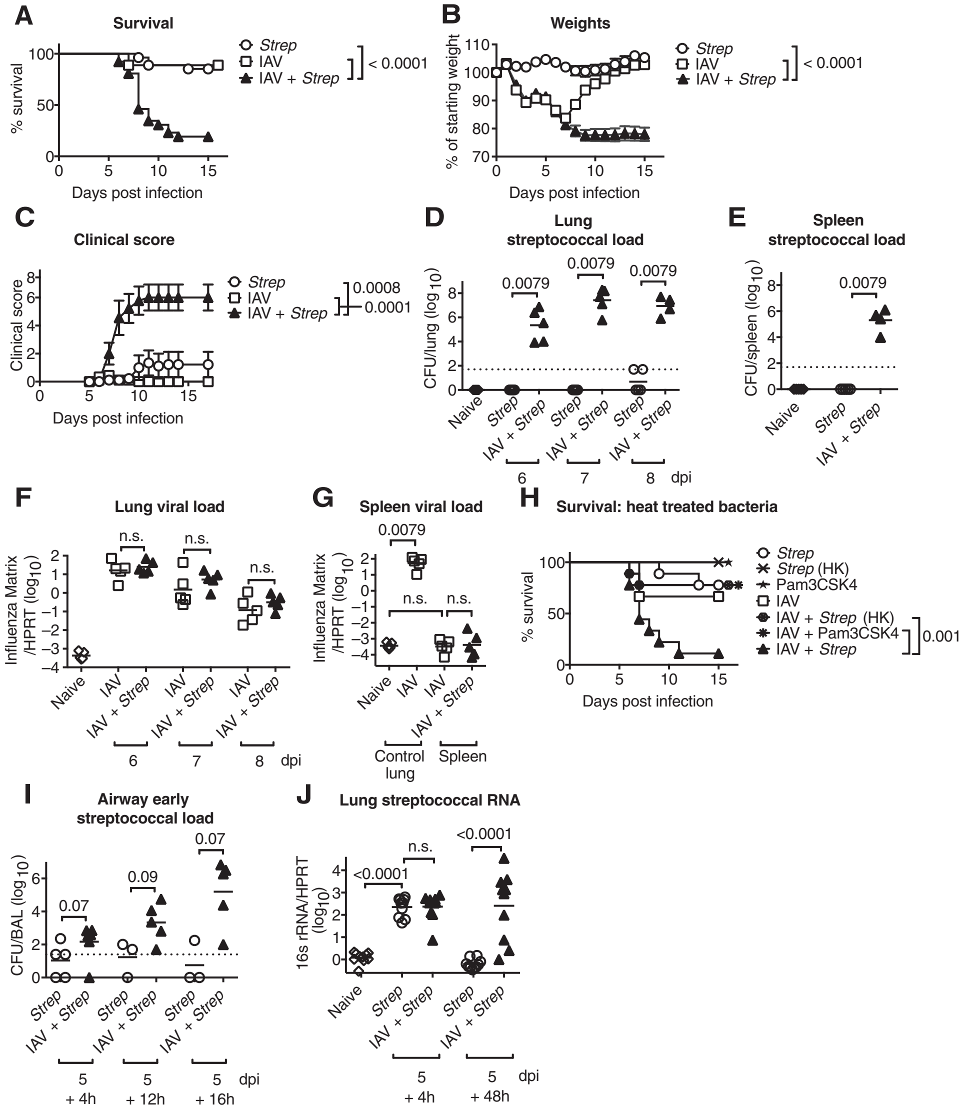

Figure EV1. Mortality in coinfection is linked to outgrowth of live bacteria.

A–C Mortality (A), weights (B) and clinical scores (C) following infection with 8×10^3 $TCID_{50}$/30 µl IAV X31, 2×10^7 CFU/30 µl *S. pneumoniae* D39 or mock (PBS) (data shown are pooled from four independent experiments, n = 6–9; this dosing regimen hereafter referred to as "high dose"; for clarity, means shown include euthanized mice at endpoint weight or clinical score).

D, E Pneumococcal load in the lung (D) from 6 to 8 dpi and in the spleen (E) at 8 dpi during low-dose coinfection (dotted line indicates detection limit, n = 4–5).

F Quantitative PCR for influenza matrix RNA in the lung during high-dose coinfection from 6 to 8 dpi (n = 5).

G Quantitative PCR for influenza matrix RNA in the spleen at 8 dpi during high-dose coinfection, compared to influenza-infected positive control lung (6 dpi 8×10^3 $TCID_{50}$ IAV) (n = 5).

H Mortality following high-dose coinfection with live *S. pneumoniae*, heat-killed *S. pneumoniae* or Pam3CSK4 (15 µg) (representative of two independent experiments, n = 6–9).

I Pneumococcal load in the airways early during high-dose coinfection from 5 dpi + 4 h to 5 dpi + 16 h (dotted line indicates detection limit, n = 5).

J Quantitative PCR for pneumococcal 16 s rRNA in the lung during high-dose coinfection from 5 dpi+4 h to 5 dpi + 48 h (n = 10).

Data information: Data are displayed as percentage survival (mortality), geometric means (viral and bacterial loads, bacterial RNA) or arithmetic means ± SEM (weights and clinical scores). Significance was assessed by Mann–Whitney *U*-test (viral and bacterial loads, bacterial RNA), two-way ANOVA (weights and clinical scores) or log-rank (Mantel–Cox) test (mortality). n.s. = not significant.

© 2015 Francis Crick Institute

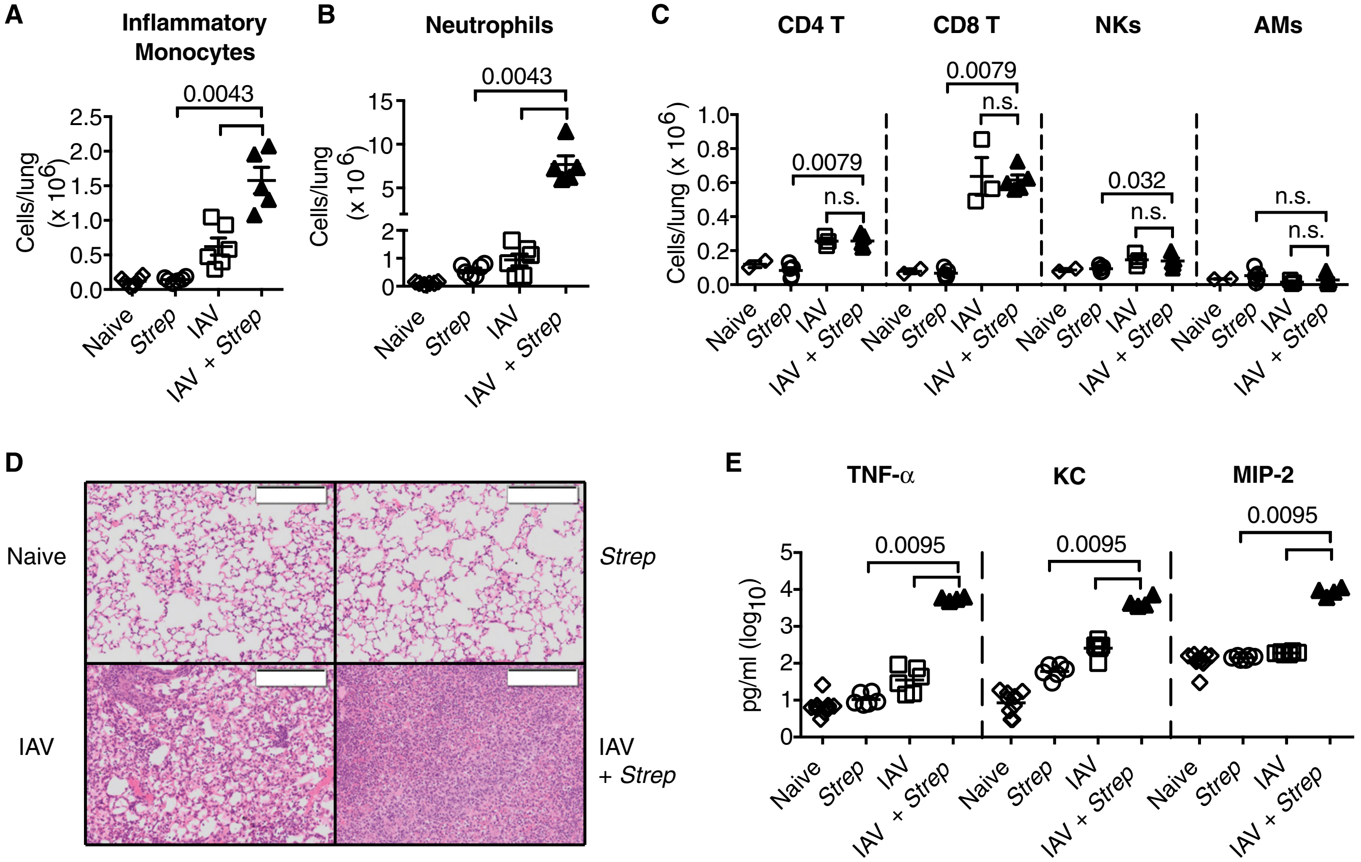

Figure EV2. Quantification of cells and cytokines during high-dose coinfection.

A, B Quantification of inflammatory monocytes (A) and neutrophils (B) during high-dose coinfection at 7 dpi by flow cytometry (data shown are pooled from two independent experiments, n = 2–3).

C Quantification of CD4 T cells ($CD3^+CD4^+$), CD8 T cells ($CD3^+CD8^+$), NK cells ($CD3^-CD4^-CD8^-NKp46^+$) and alveolar macrophages during high-dose coinfection at 7 dpi (n = 2–5).

D H&E staining of lung tissue sections at 8 dpi during high-dose coinfection. Scale bar indicates 200 μm (n = 2–3).

E Multiplex quantification of TNF-α, KC and MIP2 in the airways at 7 dpi during high-dose coinfection (n = 2–6).

Data information: Data are displayed as arithmetic means ± SEM. Significance was assessed by Mann–Whitney U-test (viral and bacterial loads, bacterial RNA). n.s. = not significant.

 © 2015 Francis Crick Institute

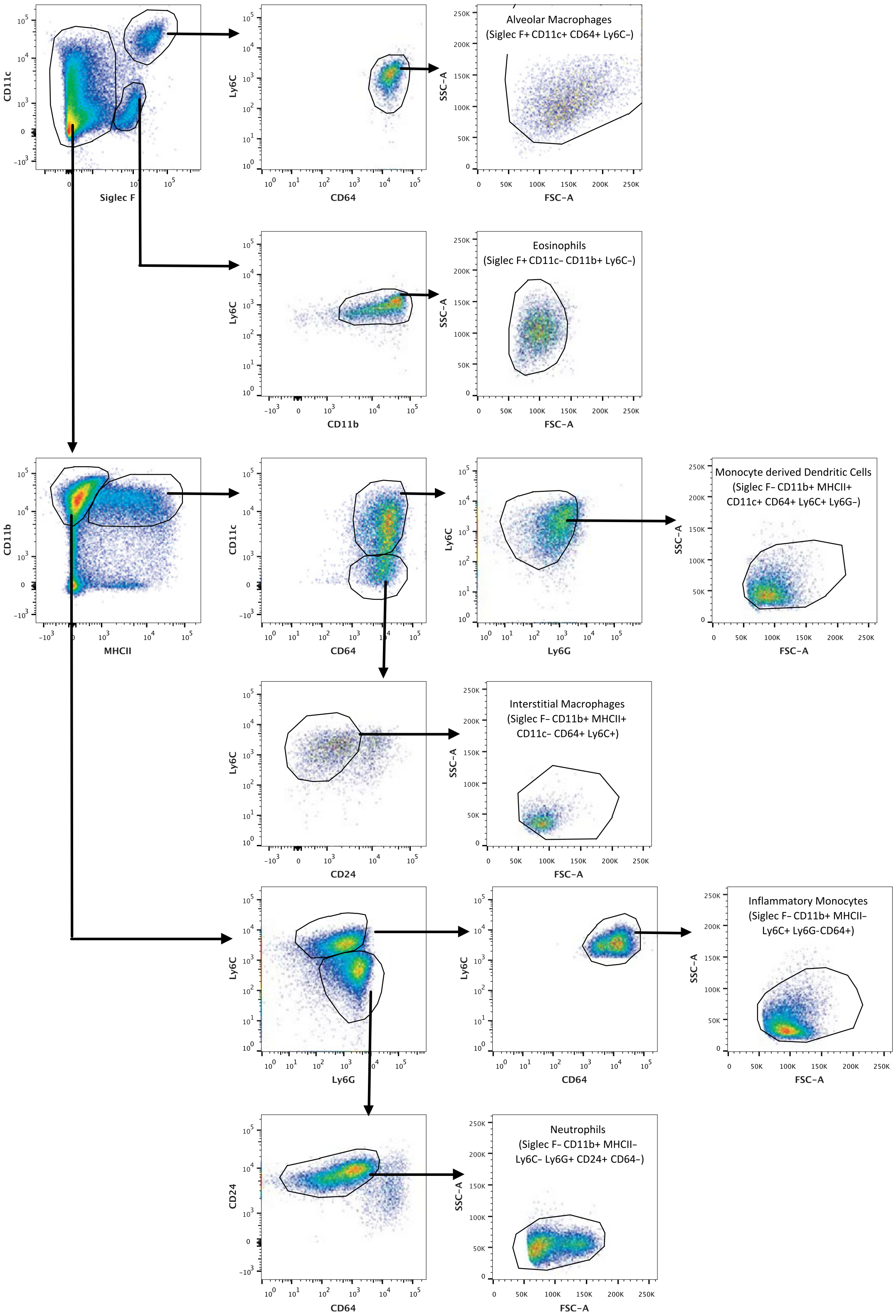

Figure EV3. Myeloid flow cytometry gating strategy.

Flow cytometry gating strategy used for myeloid cells (neutrophils, alveolar macrophages, inflammatory monocytes and inflammatory monocyte-derived cells); pre-gated for live cells (Death Stain^{-} and exclusion of debris by size). Representative 5 dpi wild-type non-lavaged whole lung shown (8×10^3 $TCID_{50}$) ($n = 4$).

© 2015 Francis Crick Institute

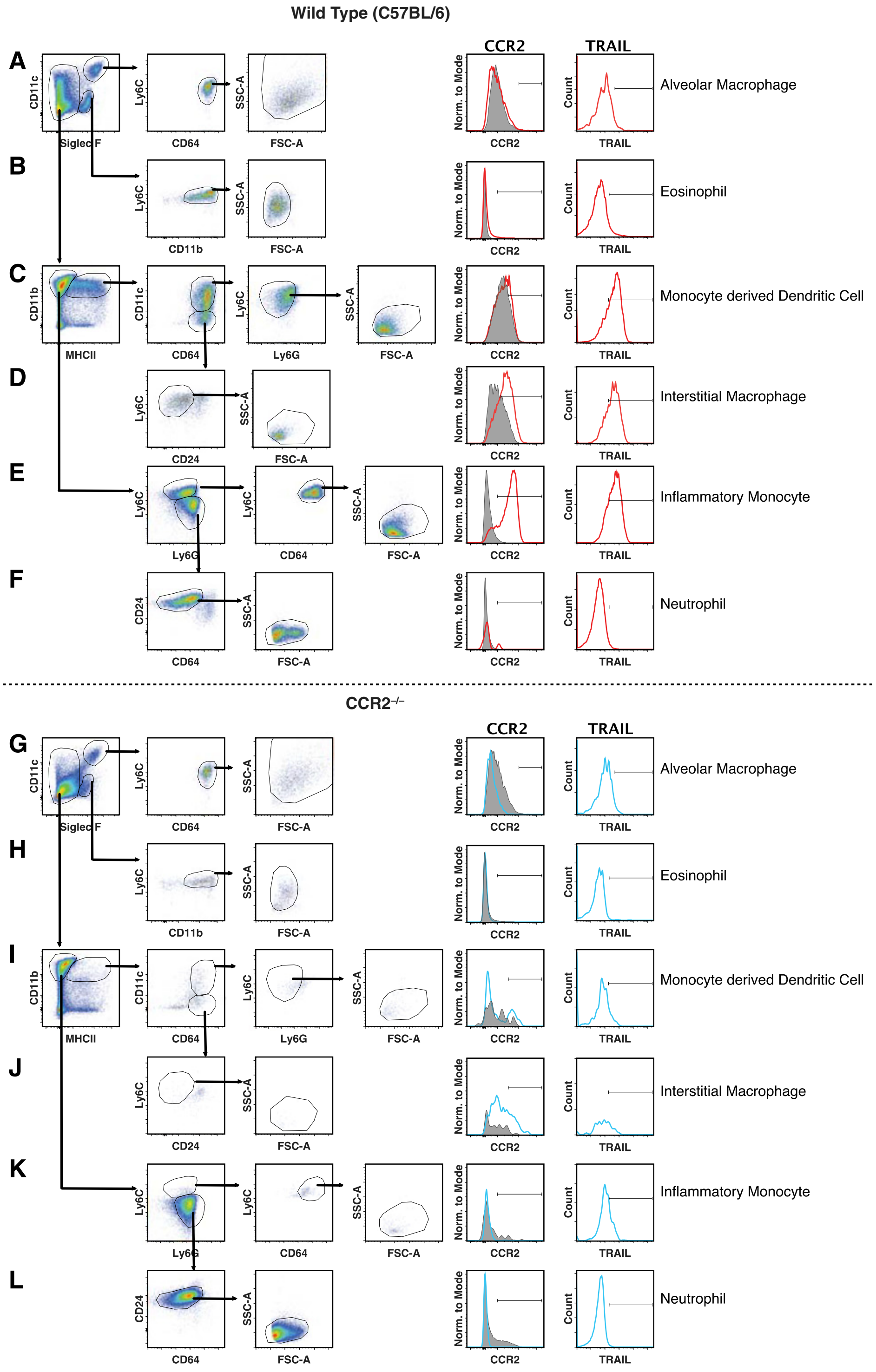

Figure EV4. Monocytes and monocyte-related cells express TRAIL and are absent in CCR2$^{-/-}$ mice.

A–F CCR2 and TRAIL expression in different myeloid cell populations as assessed by flow cytometry; pre-gated for live cells (Death Stain^{-} and exclusion of debris by size). Representative 5 dpi wild-type non-lavaged whole lung shown (8 × 10^3 $TCID_{50}$) (*n* = 4; note this lung is the same as shown in Fig EV3).

G–L CCR2 and TRAIL expression in different myeloid cell populations as assessed by flow cytometry. Representative 5 dpi non-lavaged whole CCR2$^{-/-}$ lung shown (8 × 10^3 $TCID_{50}$) (*n* = 4).

Data information: Gating of each myeloid population is shown in the left panels, and histograms of CCR2 and TRAIL expression are shown on the right. Grey histogram represents unstained, and coloured histogram represents stained.

© 2015 Francis Crick Institute

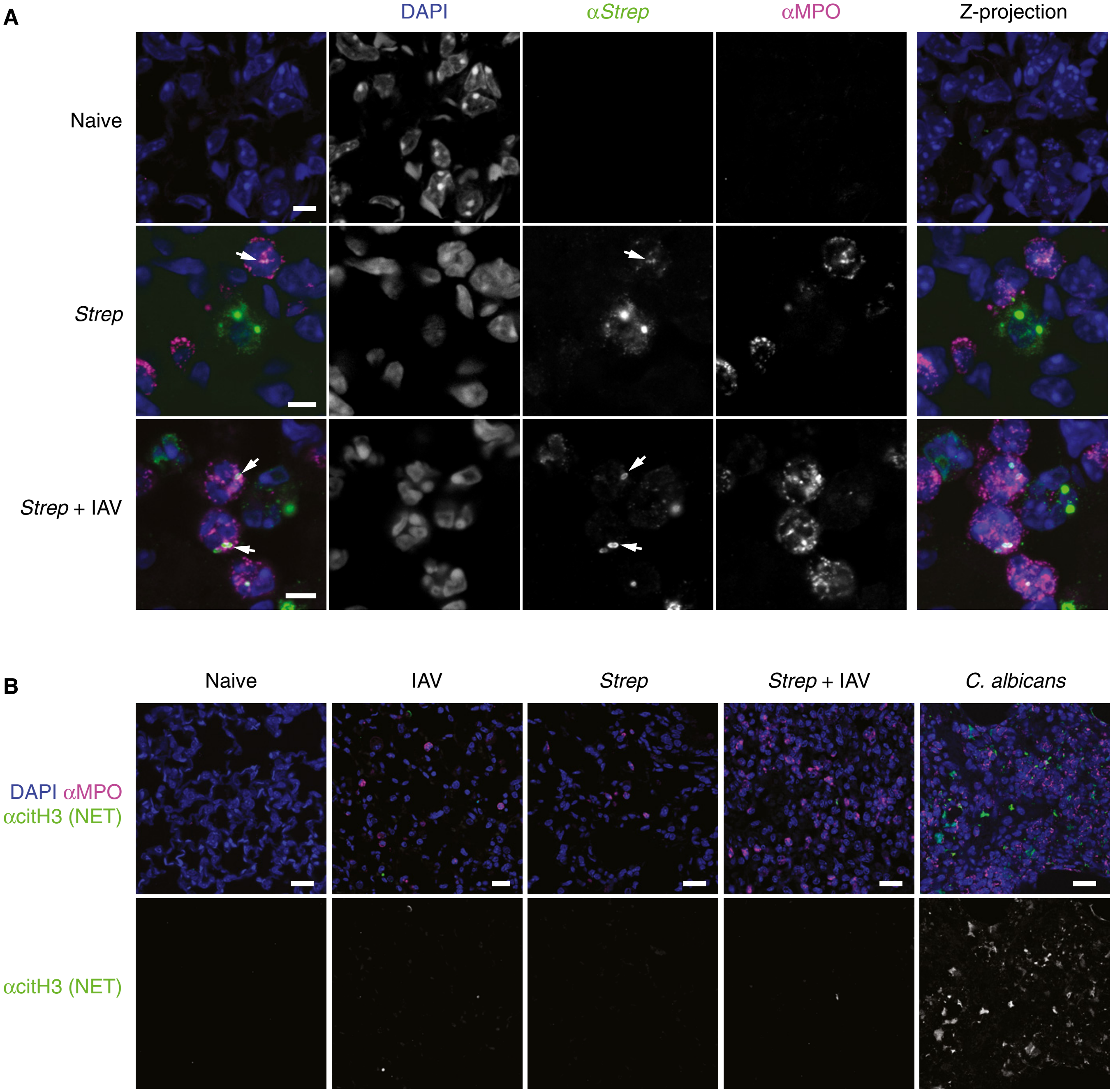

Figure EV5. Neutrophils phagocytose streptococci but do not produce NETs during coinfection.

A Confocal microscopy of lung tissue sections at 8 dpi during high-dose coinfection stained for cell nuclei (DAPI), streptococci (α*Strep*) and neutrophils (αMPO). Black text indicates infection condition; coloured text indicates staining. Right column shows the Z-projection of 10 individual focal planes; other columns show a single plane. Arrows indicate bacteria phagocytosed by neutrophils. Scale bars indicate 5 μm (n = 3).

B Confocal microscopy of lung tissue sections at 8 dpi during high-dose coinfection (or during *C. albicans* infection as positive control) stained for cell nuclei (DAPI), neutrophils (αMPO) or the NET constituent citrullinated histone H3 (αcitH3). Black text indicates infection condition; coloured text indicates staining. Scale bars indicate 20 μm (n = 3).

© 2015 Francis Crick Institute
