# Supplementary material for: TRAIL+ monocytes and monocyte-related cells cause lung damage and thereby increase susceptibility to influenza–Streptococcus pneumoniae coinfection
Source: EMBO Rep. 2015 Aug 18;16(9):1203–18. doi: 10.15252/embr.201540473 (PMC4576987; doi:10.15252/embr.201540473)
Supplement: Supplementary file 2 [file embr0016-1203-sd2.pdf]

Table EV1 - Airway cytokine levels during coinfection.

Multiplex quantification of airway cytokines at 7dpi during high dose coinfection (data shown is pooled from 2 independent experiments, n=2-3).

Data information: Data shown as arithmetic means. Fold change vs. single infections is the geometric mean of fold change IAV + *Strep* vs. *Strep* and fold change IAV + *Strep* vs. IAV. Significance assessed by Mann-Whitney test. *p<0.05 IAV + *Strep* vs *Strep*; †p<0.05 IAV + *Strep* vs. IAV.

Table EV 1

	Cytokine at 7dpi (pg/ml)				Fold change vs. single infections
	Naïve	*Strep*	IAV	IAV + *Strep*	
**TNF-α**	8	11	45	5682 *,†	256
**MIP-1α**	61	321	1338	87599 *,†	134
**RANTES**	12	10	16	1517 *,†	119
**G-CSF**	31	1002	4864	183797 *,†	83
**IL-10**	2	4	137	1858 *,†	80
**IL-6**	5	101	9325	69182 *,†	71
**MIP-1β**	101	228	1139	30002 *,†	59
**IP-10**	10	2006	10511	253229 *,†	55
**MIP-2**	128	143	190	8962 *,†	54
**IFN-γ**	26	26	3591	14232 *,†	47
**KC**	10	65	281	4778 *,†	36
**MIG**	26	671	10622	75288 *,†	28
**MCP-1**	63	506	1828	21613 *,†	22
**LIF**	2	3	311	431 *	13
**M-CSF**	11	15	48	349 *,†	13
**Eotaxin**	14	18	565	830 *	8
**VEGF**	15	65	86	426 *,†	6
**IL-1α**	137	130	36	366 *,†	5
**IL-15**	16	14	7	48 *,†	5
**IL-1β**	84	96	90	441 *,†	5
**IL-17**	2	3	16	34 *	5
**LIX**	222	176	174	717	4
**IL-5**	4	9	429	225 *	4
**GM-CSF**	32	41	37	75	2
**IL-12 (p40)**	21	50	22	57 †	2

*$p<0.05$ IAV + *Strep* vs *Strep*
†$p<0.05$ IAV + *Strep* vs. IAV.
IL-2, IL-3, IL-4, IL-7, IL-12 (p70) and IL-13 excluded as all means <20pg/ml
