# Supplementary material for: TRAIL+ monocytes and monocyte-related cells cause lung damage and thereby increase susceptibility to influenza–Streptococcus pneumoniae coinfection
Source: EMBO Rep. 2015 Aug 18;16(9):1203–18. doi: 10.15252/embr.201540473 (PMC4576987; doi:10.15252/embr.201540473)
Supplement: Supplementary file 3 [file embr0016-1203-sd3.pdf]

EMBO
reports

Manuscript EMBOR-2015-40473

TRAIL+ monocytes and monocyte-related cells cause lung damage and thereby increase susceptibility to influenza-S. pneumoniae coinfection

Gregory T. Ellis, Sophia Davidson, Stefania Crotta, Nora Branzk, Venizelos Papayannopoulos and Andreas Wack

Corresponding author: Andreas Wack, Francis Crick Institute

Review timeline:

Transfer date:	31 March 2015
Correspondence:	31 March 2015
Correspondence:	10 April 2015
Editorial Decision:	14 April 2015
Revision received:	05 June 2015
Editorial Decision:	01 July 2015
Revision received:	10 July 2015
Accepted:	10 July 2015

Editor: Nonia Pariente

Transaction Report:

(Note: With the exception of the correction of typographical or spelling errors that could be a source of ambiguity, letters and reports are not edited. The original formatting of letters and referee reports may not be reflected in this compilation.)

Transfer Note:

Please note that this manuscript was originally submitted to The EMBO Journal, where it was peer-reviewed. It was then transferred to EMBO reports with the original referees comments attached. (Please see below)

Original referees' comments – The EMBO Journal

Referee #1:

This manuscript presents three independent observations: 1) CCR2-mediated TRAIL+ monocyte recruitment is detrimental to the host; 2) neutrophils contribute to resistance against secondary bacterial infection; and 3) TNF-alpha is also protective during co-infection.

1. Fig 1 essentially repeats what others have consistently seen during influenza -pneumococcus co-infection. It is not obvious why it is felt that those findings need to be shown again in this manuscript.

2. It has already been demonstrated by others that in the absence of CCR2 or TRAIL there is decreased inflammation from influenza virus infection. Like the current study, the previous papers reported no significant effect of CCR2 or TRAIL on influenza viral titers. If CCR2 KO mice and mice treated with anti-TRAIL antibody show diminished inflammation as already reported, decreased mortality and morbidity following bacterial co-infection as seen here, is to be expected.
3. Was TRAIL expression analyzed on NK and T cells? It has been shown that influenza infection upregulates TRAIL expression on both NK and T cells (JIV, Ishikawa et al, 2005; JI, Brincks et al, 2008). Moreover, CD8+ T cells are known to be the primary effector cells responsible for the killing of flu infected epithelial cells, so even in the absence of TRIAL+ monocytes in CCR2-/- mice, significant killing of virally infected epithelial cells will still occur (and in fact, it does occur as shown in Fig 2F), yet CCR2 deficient mice show decreased susceptibility to secondary bacterial infection. Is this because there is a threshold effect of epithelial cell death for the increased susceptibility to secondary bacterial infection and this threshold is not reached in the absence of TRAIL+ monocytes in CCR2 deficient mice? It would be informative to quantitatively analyze by flow cytometry the absolute numbers of apoptotic/necrotic epithelial cells in WT and CCR2-/- mice following flu infection. If the authors are correct, the numbers of apoptotic/necrotic epithelial cell should be reduced in CCR2-/- deficient mice.
4. What is the influence of TRAIL and CCR2 on expression of types 1 and 2 interferon? As stated above, T and NK cells express TRAIL, these cells are a major source of IFN expression, and the IFNs have been shown to be the primary mediators of co-infection susceptibility.
5. Figs. 4-5, dealing with TNF-alpha and neutrophils are disconnected from the rest of the manuscript. It has been previously reported that TNF and neutrophils are required for protection against pneumococcal infection. The authors state that in the current study, they were only required for protection from co-infection. There is no further explanation but it is highly likely that the single bacterial infection in the current study was at such a low dose that the mice were fully protected from death by alveolar macrophage-mediated clearance and additional neutrophil-mediated protection was not required. Only during co-infection when there was large bacterial outgrowth would these innate mediators of protection be required. Thus, the explanation for the authors' observations is relatively trivial.
6. In the results section, FigE6A (page 13) is presented in the text before FigE5B,C and D. Reference to the figures should appear in the main text in numerical order.

Referee #2:

Comments for the authors of The EMBO Journal manuscript EMBOJ-2015-91416:
The authors of The EMBO Journal Manuscript: "TRAIL+ monocytes induce lung damage increasing susceptibility to influenza-S. pneumoniae coinfection", present some very interesting results that evaluate the balance of pathogenesis or protection in the context of a coinfection. Specifically, the authors have identified the TRAIL+ monocytes as key cells that influence coinfection outcomes, based on whether they are allowed access to the lungs early during the viral phase of infection. This TRAIL-mediated lung damage is critical for allowing neutrophils, under the control of TNF-α to limit bacterial outgrowth from the lungs. This study includes a large set of results that lead the authors to their conclusions. However, as presented, I do have some points of concern that I would like the authors to consider.
General Comments:
1. The data presented allow the authors to tell a very nice story, especially as it relates to the early vs. late anti-TRAIL therapy during a coinfection. I am also very interested in the pathogenesis vs. protection components of these studies. However, I am concerned with the interpretation of the results as it relates to CCR2-mediated recruitment of cells into the lungs. Since the lethal aspect of the coinfection was linked to the outgrowth of bacteria from the lungs, could another interpretation of the CCR2-/- data be that the effector cells remain in the periphery where they could more effectively eliminate the pathogen without the detrimental effects of inflammation within the lung? Maybe preventing TRAIL+ monocytes from entering the lung could be beneficial for the host's ability to handle the secondary bacterial invader. If the authors could comment on this aspect of their model, it would be appreciated.
2. Some of the statistically significant differences reported did not appear to be biologically significant. This is most notable in Figure 4E where neutrophil depletion did not greatly affect bacteria within the lung. If the authors want to put forth the argument that increased survival in this model (Figure 4D) is due to more rapid clearance in the presence of neutrophils, then data from Day

9 and/or Day 10 should also be shown.
3. I would like to see a visual model added to the manuscript that directly shows the interpretation of the results that the authors present. At this time, I was unclear as to how the authors envision the three factors studies (CCR2, TRAIL, and TNF-α) work together to prevent death after coinfection. This was particularly difficult to visualize since the timing of anti-TRAIL treatment affected the outcomes, and this was based on whether the anti-TRAIL was delivered early (during the viral phase) or late (during the bacterial phase) of the infection.
4. I was less enthusiastic about the studies performed with purified neutrophils from the lungs of mice (Figure 4A-C). In particular, I think there are additional aspects of neutrophil function that need to be evaluated in the actual animal, rather than through removal of cells from the lungs, and evaluation in culture. It seems that the lack of a difference in neutrophil function that the authors report could be due to the fact that these cells were removed from the environment of the infected lung. I would prefer to see an attempt to characterize the neutrophils within the lung environment. This could be done by looking at NET formation, myeloperoxidase, defensins, lactoferrin, gelatinase/MMP9, and/or phagocytic neutrophils present in tissue sections (with immunofluorescent staining where appropriate).
5. Similar to comment 4, I was curious how the mice in the Ly6G-treated group that were infected with Strep alone (Figure 4D) performed within the clinical score evaluation (Figure E1B). Since there were scores for the Strep alone mice in the figure presented, does the absence of neutrophils in these mice increase the illness observed?
Specific Comments:
1. In the Introduction, the authors state that influenza virus infections are frequently complicated by secondary bacterial coinfections. It seems to me that this could be re-worded to state that deaths after influenza virus infections are frequently due to complications associated with secondary bacterial infections.
2. In the third paragraph of the Introduction (Page 5, lines 9-11), I had a tough time understanding the point of this statement by the authors. Please clarify this statement.
3. In the Results section (Page 9, line 7), the panel referenced (Figure E1B) shows that infection with S. pneumoniae alone shows a minimal clinical sign, rather than no clinical sign, and the authors should mention what was observed that led to the assigned scores given.

Referee #3:

In this manuscript, the authors developed a coinfection (IAV/S. pneumoniae) model of moderate severity to analyze mechanisms leading to bacterial colonization and bacterial outgrowth. The originality of the paper is to "separate" these two events and to mimic situation observed during regular influenza seasons (mild influenza). In this experimental system, the authors show (i) that CCR2 (probably by recruiting inflammatory monocytes) exacerbates susceptibility to bacterial infection by promoting tissue damage (through TRAIL expression) and (ii) that neutrophils protect against bacterial outgrowth in IAV-experienced animals, possibly through TNFa release. The subject is of great interest because clinically relevant, the model described is well controlled and the manuscript adds new information in the field. However, this manuscript suffers from a lack of mechanistic insights explaining in more details the role of inflammatory monocytes and neutrophils in bacterial superinfection. Below are other issues that, if addressed, might improve the quality of the manuscript.

The first part dedicated to the acute severity coinfection model (high dose) is too long and too descriptive. This part does not really provide new information. Moreover, the dose used (2x10e7 bacteria) does not really correspond to a "physiological" dose. This is enormous. For the rest of the study, the authors concentrate on the moderate ("low dose") severity coinfection model. Here too, the dose (2x10e5) is quite important (D39 has a low infectivity potential in the mouse system) and might raise concern about the significance of the data shown in the manuscript. In Fig. 1B, the authors claimed (page 9) that viruses are cleared but this is not the case (the viral load is just reduced). This should be reworded.

In Fig. 2 (page 11), there is an improved control of bacterial outgrowth and survival in CCR2 deficient animals. Is it associated with reduced recruitment of inflammatory monocytes in the lungs? The authors claim that inflammatory monocytes are involved in CCR2-mediated bacterial superinfection but this is not shown. The authors state that the numbers of CFU are decreased in

spleen and brain (page 12) but this is not shown.
To further demonstrate that CCR2 plays a role in coinfection in this model, a neutralizing Ab should be used. WT and CCR2 KO littermates were not used in Fig. 2. Moreover, this strategy might give additional information (e.g. treatment at early and later stage of influenza, as in Fig. 3D).
Concerning the gating strategy shown in Fig. E2, the CD11b/CD11c labeling is not very discriminative (it is difficult to visualize CD11c-positive dendritic cells on the dot plot). The authors should use an anti-MHC class II Ab do make sure that there is no DCs in the inflammatory monocytes population. The authors might also use an anti-Siglec F Ab to label alveolar macrophages. What is the percentage of CCR2-positive cells within the inflammatory monocytes population?

Judging by Fig. 3A (left panels), approximately 50% of TRAIL-positive cells are inflammatory monocytes. Do the authors know the nature of other TRAIL-positive cells? A more complete analysis should be done. This is important since the effect is probably not fully mediated by inflammatory monocytes. It might be interesting to show that TRAIL expression on inflammatory monocytes is involved in epithelial cell (as well as other cells) apoptosis. Do the authors have access to conditional knock-out mice? Usually, the anti-CD64 Ab is used to label inflammatory monocytes (Langlet et al. 2012). What is the percentage of CD64-positive "inflammatory monocytes" in flu infected mice?

In Fig. 4, depletion of neutrophils should be shown. Neutrophil depletion has no effect in mice only infected with Sp. I guess this is due to the low infectivity rate of D39 and that macrophages are implicated in this setting.

It might be interesting to determine the source of TNFa.

To conclude, this is an interesting paper and the model used by the authors is interesting as it might mimic mild influenza/bacterial superinfection (although this can be debated; e.g. the high dose of bacteria used). The idea to separate events involved in bacterial colonization versus bacterial outgrowth is also well appreciated. Additional work is however needed to improve the quality of the manuscript.

Correspondence - editor EMBO reports — 31 March 2015

I have now had a chance to read the EMBOJ referee reports and, before making a decision, I would be interested to know how you would plan to address the comments. Although all referees find the topic of interest (as do I), they raise concerns about the novelty, conclusiveness and overall insight provided. Some of the results could be moved to the supplement, to give you space to strengthen the rest of the work. Although some issues -such as analyzing the effect of TRAIL monocyte expression on epithelial cells using conditional knock-outs and finding the source of TNFalpha- would be beyond the scope of this study, I think most of them should be addressed to some extent.

In order to gauge how the study would fare here once revised, I would need a clearer picture of what a revision would entail. Can you thus send us a detailed point-by-point response to the referee concerns?

We now publish also full-length format articles, so the length would not be a problem.

I look forward to hearing from you.

Correspondence – authors's reply — 10 April 2015

Attached is our detailed reply to referees' concerns, please have a look. Sorry for being a bit lengthy, but we thought we should give as detailed a response as we could - if this is too long or to make your life easier anyway I am happy to also send you a condensed list of experiments underway and planned to address each point. I think (and we hope you will agree) that we can cover all points in a satisfactory manner.
Thanks for your interest and looking forward to your comments.

© European Molecular Biology Organization

<u>Addressing Referees' Comments – "TRAIL+ monocytes induce lung damage increasing susceptibility to influenza–S. pneumoniae coinfection" by Ellis et al considered for EMBO Reports</u>

Referees' comments are in plain text, ***comments from the authors are in bold and italicized.***

General remark to the editor:

Before responding to the referees' suggestions below in detail, there are two points that we would like to bring to the attention of the editor:

1. We have calibrated our experimental system to pathogen doses low enough to give zero mortality and very low morbidity by single infection, but leading to 50% mortality and massive immune responses in coinfection. We think this is a very useful model as it is closer to the clinical situation during influenza seasons, reflects the constant low-dose polymicrobial exposure of humans better, and allows us to study interventions that are either harmful or protective. Given the very low impact of the single infections, we perceive coinfection as a disease separate from the individual infections, with divergent, maybe opposing cues given to the immune system at different phases of coinfection, and with unpredictable disease course and outcome. While it is legitimate and useful to compare this disease model to (mostly, if not always, severe or lethal) single infection models, we think it is wrong to then go on to conclude that similarity to single severe infection removes novelty or relevance from our data, and inversely, that results contradicting single infection data are difficult to explain or render the model less valid. Exposing the lung to a combination of two mild but divergent stimuli is simply a disease that is different from a single severe infection.

2. Part of the price we pay for using this low dose coinfection model is that results spread and don't look as nice a severe coinfection with 100% mortality (as directly compared in fig. 2B). While this allows us to learn more (fig. 2B and others), it means that statistical significance is reached only with high numbers of animals. We routinely use 9 mice per infection group and often pool experiments to obtain statistical significance. Running a full experiment twice, with single and co-infection, therefore translates into 2 x 3 x 9 = 54 mice; this number doubles or trebles when different genotypes or regimens are compared. We think this is ethically defensible as our model is closer to clinical reality and allows us a better understanding of coinfection, but we would like this logistical burden to be kept in mind when additional experiments or the use of littermate controls are discussed below.

And now the replies to the individual reviewers' comments:

Referee #1:
This manuscript presents three independent observations: 1) CCR2-mediated TRAIL+ monocyte recruitment is detrimental to the host; 2) neutrophils contribute to resistance

against secondary bacterial infection; and 3) TNF-alpha is also protective during coinfection.

Although the reviewer classifies these observations as independent, we feel that presented together they provide a clear narrative of successive events in coinfection.

1. Fig 1 essentially repeats what others have consistently seen during influenza - pneumococcus co-infection. It is not obvious why it is felt that those findings need to be shown again in this manuscript.

It is correct that a number of previous studies show many of the aspects we show here. Figure 1 establishes the model used and gives a rationale for further analysis of monocyte, neutrophil and TNFa effects, as these factors clearly dominate the immune response. However, this figure can be moved to supplemental figures or removed entirely if the editor or reviewers deem this right. However, some previous studies suggest influenza-mediated immune impairment as a mechanism of coinfection, with reductions in TNF-α or IL-17 (Sun/Metzger, Nat Med, 2008; Li/Moran, J Virol, 2012) or neutrophils (Shahangian/Deng, JCI, 2010). Therefore it is relevant that, as shown in Figure 1, we do not observe reductions in these factors in our model.

Figure 1, which uses a high dose, aims to profile high-mortality coinfection clearly, as the disease outcome is consistent within groups. Low dose coinfection used in later figures attempts to give a scenario closer to the clinic and where both pathogenic and protective interventions can be detected. Profiling in low dose coinfection gives similar trends to high dose, but less clear and often not reaching statistical significance, as there is (by design) greater spread in disease course and outcome within groups (see fig. 2B). For example:

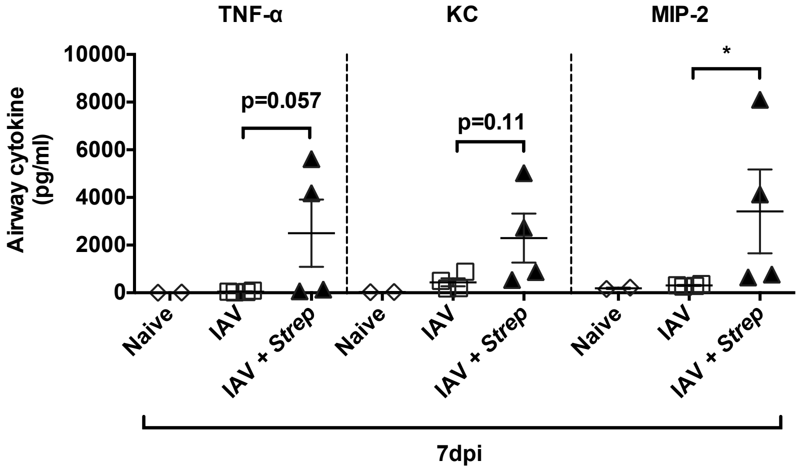

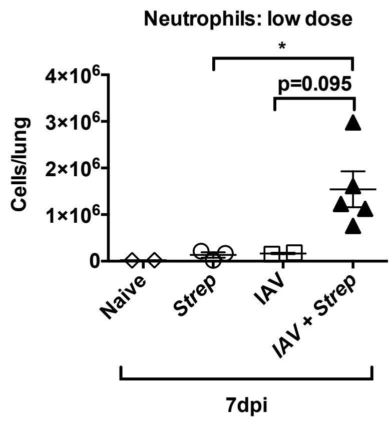

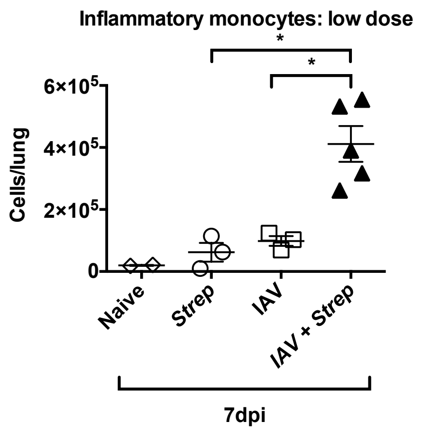

If it is felt that statistically significant data needs to be provided for the low dose coinfection, we can repeat these experiments and will eventually reach significance. We feel however that it is legitimate (and useful) to profile the immune response at doses giving more consistent disease outcome, since we confirm here that our findings in low dose coinfection show the same trends for all parameters assessed.

2. It has already been demonstrated by others that in the absence of CCR2 or TRAIL there is decreased inflammation from influenza virus infection. Like the current study, the previous papers reported no significant effect of CCR2 or TRAIL on influenza viral titers. If CCR2 KO mice and mice treated with anti-TRAIL antibody show diminished inflammation as already reported, decreased mortality and morbidity following bacterial co-infection as seen here, is to be expected.

Here Referee 1 argues that as the role of TRAIL and CCR2-dependent monocytes in causing damage and being on balance harmful has been previously investigated in severe influenza, that it can be implied that they will perform a similar function in coinfection. There are several points to be made which address this:

1. ***Coinfection is a clinically relevant, distinct disease context from influenza alone, and therefore the role of TRAIL and CCR2-dependent monocytes in coinfection merits separate investigation, and results are to be considered novel.***
2. ***The net effect of CCR2-dependent monocytes and TRAIL as protective or harmful in influenza has not been conclusively established. Some studies report that they cause damage and are net harmful (e.g. Herold/Lohmeyer, J Exp Med, 2008). However, others show a protective or harmful role depending on influenza severity (e.g. Aldridge/Thomas, PNAS, 2008). Also, these papers use highly severe or lethal flu models (80% mortality in Herold, 50-90% mortality in Aldridge, depending on the model), while flu in our model does not cause mortality, and no differences are found in single infections between wt and CCR2 KO mice. The referee points out correctly that "Like the current study, the previous papers reported no significant effect of CCR2 or TRAIL on influenza viral titers", but fails to report that there were massive changes in weight loss and mortality in the severe influenza infections, which we don't find in our single influenza infection (mortality is reported in fig. 3C, weight loss and clinical scores can be added for single and for coinfections). It is therefore not correct to simply extrapolate from published data to what we report here.***
3. ***The second pathogen in coinfection - S. pneumoniae - must also be considered; as in influenza, there is not a clear consensus on their role. It has been reported in many studies of more severe S. pneumoniae infections that TRAIL and CCR2 are protective (Davis/Weiser, JCI, 2011; Winter/Maus JI 2007, 2009; Steinwede/Maus, J Exp Med, 2012), again indicating that single infection data does not predict coinfection outcome; however, other studies have found no effect in S. pneumoniae infection of the brain (Mildner/Prinz, J Imm, 2008).***

4. ***Given the contradicting results found in influenza and Pneumococcus infections, it is neither trivial nor in fact possible to extrapolate the role of TRAIL and CCR2-dependent monocytes from single infections to coinfection.***

3. Was TRAIL expression analyzed on NK and T cells? It has been shown that influenza infection upregulates TRAIL expression on both NK and T cells (JIV, Ishikawa et al, 2005; JI, Brincks et al, 2008). Moreover, CD8+ T cells are known to be the primary effector cells responsible for the killing of flu infected epithelial cells, so even in the absence of TRIAL+ monocytes in CCR2-/- mice, significant killing of virally infected epithelial cells will still occur (and in fact, it does occur as shown in Fig 2F), yet CCR2 deficient mice show decreased susceptibility to secondary bacterial infection.

We do not rule out the possible importance of TRAIL on CD8 or NK cells and cite one of the above studies (Brincks/Legge, J Imm, 2008) in our manuscript. We have not cited Ishikawa as they show delayed virus clearance but do not analyse changes in severity in influenza when TRAIL is blocked by an antibody. We have however stained for TRAIL on $CD8^+$ T cells and find the following picture, indicating that CD8 T cells are not a major contributor to TRAIL presence in infected lungs at the time point studied.

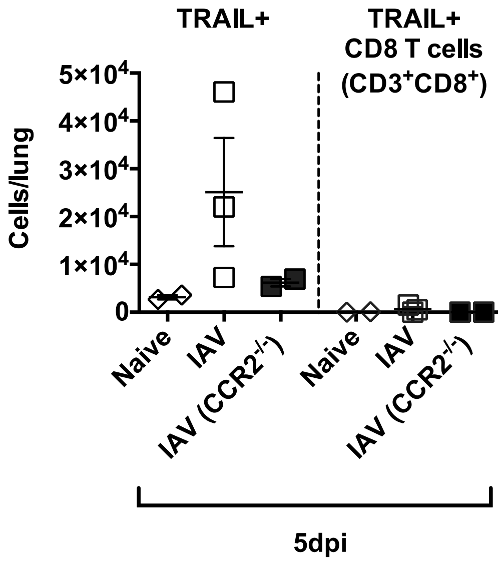

We have also started analysing the role of NK cells in this model but have not yet conclusive results for this. We detect very low levels of TRAIL on NK cells in infected wt mice and find NK cell numbers unchanged in infected CCR2 deficient mice which are protected, suggesting that the role of NK cells in this coinfection model is minor. In line with this, several papers have now shown that the role of NK cells in influenza infection is not central (Monticelli/Artis, ni 2011; Abdul-Careem/Ashkar, JID 2012), suggesting that damage in mild flu prior to bacterial superinfection is NK cell - independent. If the editor and referees deem the addition of the above-mentioned results important then we can repeat these NK cell experiments until conclusive statements concerning TRAIL expression

on NK cells and CCR2-dependence of NK cell can be reached. We suggest to repeat experiments and establish a conclusive data set on TRAIL expression on NK cells and NK cell numbers in infected wt and CCR2-/- mice at the time point where bacterial infection sets in and TRAIL blockade is protective, which would be complementary to the data set in the manuscript (fig. 3A) and the above data on CD8 T cells.

Is this because there is a threshold effect of epithelial cell death for the increased susceptibility to secondary bacterial infection and this threshold is not reached in the absence of TRAIL+ monocytes in CCR2 deficient mice? It would be informative to quantitatively analyze by flow cytometry the absolute numbers of apoptotic/necrotic epithelial cells in WT and CCR2-/- mice following flu infection. If the authors are correct, the numbers of apoptotic/necrotic epithelial cell should be reduced in CCR2-/- deficient mice. ***Yes we tend to agree, but rather than a threshold we think the bacterial outgrowth that we observe is the consequence of a singular or very few instances of bacteria crossing the epithelial barrier, and reducing epithelial damage reduces the likelihood of this relatively rare event to occur. We will analyse epithelial cell apoptosis in wt and CCR2 deficient flu-infected mice by FACS or in sections, as proposed here, even though the low-dose regimen we use may make definitive statements difficult.***

4. What is the influence of TRAIL and CCR2 on expression of types 1 and 2 interferon? As stated above, T and NK cells express TRAIL, these cells are a major source of IFN expression, and the IFNs have been shown to be the primary mediators of co-infection susceptibility.

We have looked at IFNg expression levels in CCR2$^{-/-}$ and wt mice and find no difference, and we will include this data. We will also look at IFNab levels in these mice. We have tested and will include our data showing that the anti-TRAIL mAb we use does not deplete cells, and since TRAIL has never been implicated in cell activation, there should be no changes in cytokine levels.

5. Figs. 4-5, dealing with TNF-alpha and neutrophils are disconnected from the rest of the manuscript. It has been previously reported that TNF and neutrophils are required for protection against pneumococcal infection. The authors state that in the current study, they were only required for protection from co-infection. There is no further explanation but it is highly likely that the single bacterial infection in the current study was at such a low dose that the mice were fully protected from death by alveolar macrophage-mediated clearance and additional neutrophil-mediated protection was not required. Only during co-infection when there was large bacterial outgrowth would these innate mediators of protection be required. Thus, the explanation for the authors' observations is relatively trivial.

There are several points to be addressed here:

1. ***Referee 1 states "TNF-alpha and neutrophils are disconnected to the rest of the manuscript". On the contrary, we feel that including this information provides a narrative of disease progression throughout coinfection, starting with upstream***

causes and progressing to downstream effects. It also demonstrates how different effector arms of the innate immune response are harmful at one stage of a complex infection (i.e. CCR2-dependent damage early) but protective later (i.e. neutrophils and TNF-α).

2. *Referee 1 states "neutrophils are required for protection against pneumococcal infection". Although neutrophils are well established as protective in other bacterial infections, their role in S. pneumoniae has not been as clearly defined as commonly supposed. For example, neutrophil depletion using the non-specific mAb anti-Gr-1 exacerbates bacterial loads in some cases (Sun/Metzger, Inf Imm, 2007) but reduces it in others (Marks/Pirofski, Inf Imm, 2007), or has no effect (Stegemann/Gunzer, Plos One, 2009), or neutrophil function was proposed to be blocked by influenza coinfection (McNamee & Harmsen, IaI, 2006), which we do not find in our mild infection setting. Therefore the role of neutrophils in coinfection cannot be easily implied from their role in single S. pneumoniae infection.*
3. *We completely agree with the referee that neutrophils are not required here in single bacterial infection because this infection is mild. We however disagree with referee 1's statement that the role of neutrophils as protective in coinfection is a trivial induction from their role in single infection. There is a lack of consensus over the role of neutrophils in coinfection, which our model addresses. One study implies (although does not directly confirm through depletion studies) inadequate neutrophil recruitment is a cause of coinfection susceptibility (Shahangian/Deng, JCI, 2009), while another study shows no effect of neutrophil depletion in lethal coinfection (Damjanovic/Xing, Am J Path, 2013).*
4. *Neutrophils are known to be crucial effector cells in fulminant and chronic lung inflammation as characterised by acute respiratory distress syndrome (ARDS) or chronic obstructive pulmonary disease (COPD) (e.g. Ichikawa/Imai, AJRCCM 2013; reviewed in Short/Kuiken, Lancet 2014), and it is therefore a plausible hypothesis that the high levels of neutrophils and TNF-α may cause damage during the bacterial phase of coinfection. However, we demonstrate that the net effect is protective. This is highly relevant in a study such as ours focusing on the role of immune-mediated damage in coinfection.*
5. *Although TNF-α has been shown to be protective in S. pneumoniae infection (e.g. Takashima/Yamaguchi, Inf Imm, 1997), this may be dependent on disease context (Kirby/Kaye, J Inf Dis, 2005). Furthermore it has been shown to have a harmful net effect in influenza infection (e.g. Hussell/Openshaw, Eur J Imm, 2001). Therefore showing it is protective in coinfection is a novel and clinically relevant observation; particularly as this may inform potential therapies.*
6. *The low pathogenicity of the single S. pneumoniae infection used in our system is not a flaw in the model, but a crucial element of it - we wanted to perform interventions in coinfection without perturbing the single infections greatly, as this would make interpreting results difficult. This required single infections of mild to moderate pathogenicity.*

6. In the results section, FigE6A (page 13) is presented in the text before FigE5B,C and D. Reference to the figures should appear in the main text in numerical order.

This can be easily fixed.

Referee #2:

Comments for the authors of The EMBO Journal manuscript EMBOJ-2015-91416:
The authors of The EMBO Journal Manuscript: "TRAIL+ monocytes induce lung damage increasing susceptibility to influenza-S. pneumoniae coinfection", present some very interesting results that evaluate the balance of pathogenesis or protection in the context of a coinfection. Specifically, the authors have identified the TRAIL+ monocytes as key cells that influence coinfection outcomes, based on whether they are allowed access to the lungs early during the viral phase of infection. This TRAIL-mediated lung damage is critical for allowing neutrophils, under the control of TNF-α to limit bacterial outgrowth from the lungs. This study includes a large set of results that lead the authors to their conclusions. However, as presented, I do have some points of concern that I would like the authors to consider.
General Comments:
1. The data presented allow the authors to tell a very nice story, especially as it relates to the early vs. late anti-TRAIL therapy during a coinfection. I am also very interested in the pathogenesis vs. protection components of these studies. However, I am concerned with the interpretation of the results as it relates to CCR2-mediated recruitment of cells into the lungs. Since the lethal aspect of the coinfection was linked to the outgrowth of bacteria from the lungs, could another interpretation of the CCR2-/- data be that the effector cells remain in the periphery where they could more effectively eliminate the pathogen without the detrimental effects of inflammation within the lung? Maybe preventing TRAIL+ monocytes from entering the lung could be beneficial for the host's ability to handle the secondary bacterial invader. If the authors could comment on this aspect of their model, it would be appreciated.

This is an interesting suggestion. Referee 2 speculates that CCR2 deficiency traps CCR2-dependent monocytes in the periphery, where they can control bacterial spread without causing lung damage. Prior studies using CCR2$^{-/-}$ mice (Serbina/Pamer, Nat Imm, 2006) conclude that the main block in CCR2 deficient mice is monocyte egress from the bone marrow to the periphery, less so from the periphery to the tissues. We therefore expect low numbers of monocytes in peripheral blood in our infection model. The mechanism proposed here has essentially been addressed by (Winter/Maus, JI 2009): they find that upon respiratory infection, some S.p. strains cause bacteremia in CCR2-/- but not wt mice, therefore not supporting the reviewer's hypothesis of accumulation of monocytes in the blood which would protect from bacteremia and spread. We would be happy to assess the blood for the presence of monocytes in naïve and flu infected wt and CCR2$^{-/-}$ mice but it appears that this interesting hypothesis has already been tested and ruled out.

2. Some of the statistically significant differences reported did not appear to be biologically significant. This is most notable in Figure 4E where neutrophil depletion did not greatly affect bacteria within the lung. If the authors want to put forth the argument that increased survival in this model (Figure 4D) is due to more rapid clearance in the presence of neutrophils, then data from Day 9 and/or Day 10 should also be shown.

As the y-axis in Figure 4E shows log_{10} values, the bacterial load changes by more than 100-fold, which likely explains different disease outcomes in 4D and therefore is biologically significant. In addition, a price we pay for the low pathogenicity model we use is that CFU values spread over a wide range (see fig. 4E isotype-matched control and fig. 2B). Despite this wide range, we are able to show that in the coinfected control group, one quarter of mice exhibit no bacterial load, while in neutrophil-depleted mice, all have bacteria present in the lungs (fig. 4E), which is in line with the survival differences shown in fig. 4D.

While sampling at day 9 or 10 would confirm this further, and we are prepared to do this if required, there is a practical/ethical issue - mice begin to reach the clinical endpoint for humane euthanasia at day 8 (see Figure 4D) and must be euthanized. Therefore, to sample mice at day 9 or 10 would by necessity be a sample of surviving mice, which is likely to bias the sample towards mice with a low bacterial load and confound the experiment.

3. I would like to see a visual model added to the manuscript that directly shows the interpretation of the results that the authors present. At this time, I was unclear as to how the authors envision the three factors studies (CCR2, TRAIL, and TNF-α) work together to prevent death after coinfection. This was particularly difficult to visualize since the timing of anti-TRAIL treatment affected the outcomes, and this was based on whether the anti-TRAIL was delivered early (during the viral phase) or late (during the bacterial phase) of the infection.

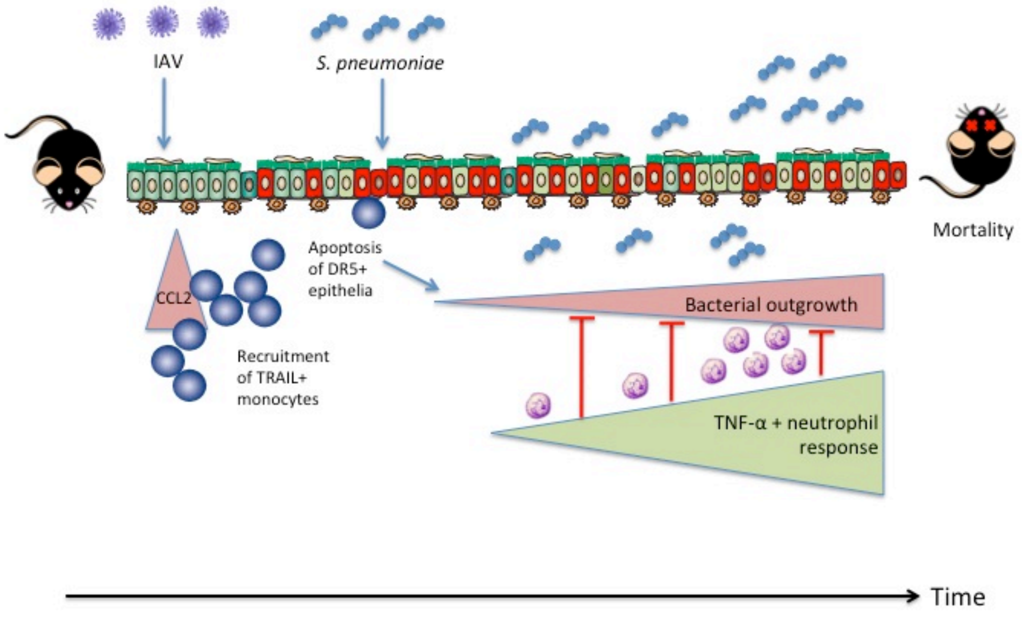

This is a depiction of the course of events according to our data: Influenza infection leads to CCR2-mediated recruitment of TRAIL+ monocytes, which cause apoptosis of DR5 expressing lung epithelia, thus permitting bacteria to invade the lung. Subsequent massive bacterial outgrowth drives a strong innate immune response dominated by TNFa and neutrophils, which contribute to bacterial control and therefore have a protective net effect. We can include a version of this figure at the end of the manuscript if deemed appropriate by the editor.

4. I was less enthusiastic about the studies performed with purified neutrophils from the lungs of mice (Figure 4A-C). In particular, I think there are additional aspects of neutrophil function that need to be evaluated in the actual animal, rather than through removal of cells from the lungs, and evaluation in culture. It seems that the lack of a difference in neutrophil function that the authors report could be due to the fact that these cells were removed from the environment of the infected lung. I would prefer to see an attempt to characterize the neutrophils within the lung environment. This could be done by looking at NET formation, myeloperoxidase, defensins, lactoferrin, gelatinase/MMP9, and/or phagocytic neutrophils present in tissue sections (with immunofluorescent staining where appropriate).

We agree that harvesting neutrophils from the lung during infection may bias the selection towards neutrophils that have recently migrated to the lung or be attached to the lung endothelium. Ex vivo assays and histology as suggested by the referee may therefore reinforce the in vitro observations and will be performed.

5. Similar to comment 4, I was curious how the mice in the Ly6G-treated group that were infected with Strep alone (Figure 4D) performed within the clinical score evaluation (Figure E1B). Since there were scores for the Strep alone mice in the figure presented, does the absence of neutrophils in these mice increase the illness observed?

We routinely assess clinical scores and are happy to provide these for the neutrophil-depleted mice.

Specific Comments:
1. In the Introduction, the authors state that influenza virus infections are frequently complicated by secondary bacterial coinfections. It seems to me that this could be re-worded to state that deaths after influenza virus infections are frequently due to complications associated with secondary bacterial infections.

Thanks for the helpful suggestion, we will change this in the revised form of the manuscript.

2. In the third paragraph of the Introduction (Page 5, lines 9-11), I had a tough time understanding the point of this statement by the authors. Please clarify this statement.

We will reword this statement in the revised form of the manuscript to make it clearer.

3. In the Results section (Page 9, line 7), the panel referenced (Figure E1B) shows that infection with S. pneumoniae alone shows a minimal clinical sign, rather than no clinical sign, and the authors should mention what was observed that led to the assigned scores given.

This will be fixed.

Referee #3:

In this manuscript, the authors developed a coinfection (IAV/S. pneumoniae) model of moderate severity to analyze mechanisms leading to bacterial colonization and bacterial outgrowth. The originality of the paper is to "separate" these two events and to mimic situation observed during regular influenza seasons (mild influenza). In this experimental system, the authors show (i) that CCR2 (probably by recruiting inflammatory monocytes) exacerbates susceptibility to bacterial infection by promoting tissue damage (through TRAIL expression) and (ii) that neutrophils protect against bacterial outgrowth in IAV-experienced animals, possibly through TNFa release. The subject is of great interest because clinically relevant, the model described is well controlled and the manuscript adds new information in the field. However, this manuscript suffers from a lack of mechanistic insights explaining in more details the role of inflammatory monocytes and neutrophils in bacterial superinfection.

It should be noted here although we show in vitro that restimulated neutrophils are functionally capable of producing TNF-α, we do not state that they are the main source of TNF-α in vivo, although this is possible. As proposed by reviewer 2, we will perform a series of ex vivo measurements and histological studies to understand better how neutrophils perform this protective effect.

Below are other issues that, if addressed, might improve the quality of the manuscript.

The first part dedicated to the acute severity coinfection model (high dose) is too long and too descriptive. This part does not really provide new information. Moreover, the dose used (2x10e7 bacteria) does not really correspond to a "physiological" dose. This is enormous. For the rest of the study, the authors concentrate on the moderate ("low dose") severity coinfection model. Here too, the dose (2x10e5) is quite important (D39 has a low infectivity potential in the mouse system) and might raise concern about the significance of the data shown in the manuscript.

Figure 1 is relevant for reasons pointed out in reply to referee 1 but can be removed or moved to supplemental data.

The dose of 2x107 CFU is consistent with that frequently used in other S. pneumoniae studies and allows us to establish that the main components under study, i.e. monocytes, neutrophils and TNFa, dominate the response. The low dose used in the intervention experiments is below what we find in many papers and yields 0% mortality, next to no weight loss and only a blip in clinical scores. As the S.p. strain used here is not an efficient coloniser (fig. E1E), we feel that this is as good as one can do it when seeking low-dose single infections.

In Fig. 1B, the authors claimed (page 9) that viruses are cleared but this is not the case (the viral load is just reduced). This should be reworded.
This is a correct observation and we will reword appropriately, saying that the time course of virus control, as measured by reduction in viral load, is identical in single infected and co-infected animals.

In Fig. 2 (page 11), there is an improved control of bacterial outgrowth and survival in CCR2 deficient animals. Is it associated with reduced recruitment of inflammatory monocytes in the lungs? The authors claim that inflammatory monocytes are involved in CCR2-mediated bacterial superinfection but this is not shown. The authors state that the numbers of CFU are decreased in spleen and brain (page 12) but this is not shown.

We have confirmed that CCR2 deficiency leads to a total lack of monocytes in the lung at several time points:

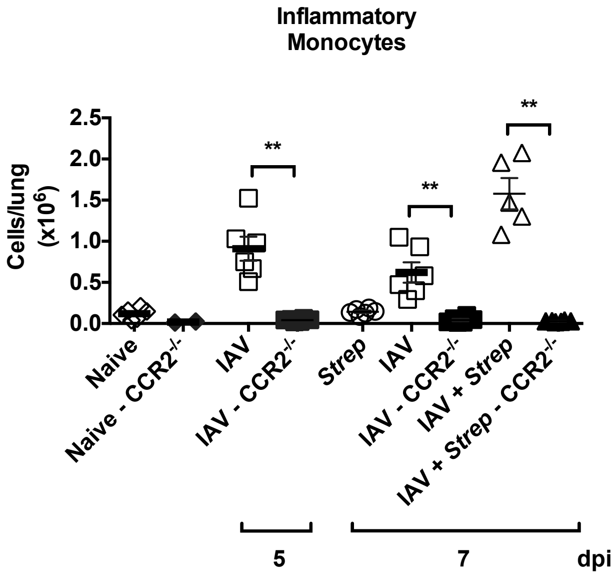

We have measured decreased spleen and brain loads (see below) and are happy to include them in the manuscript:

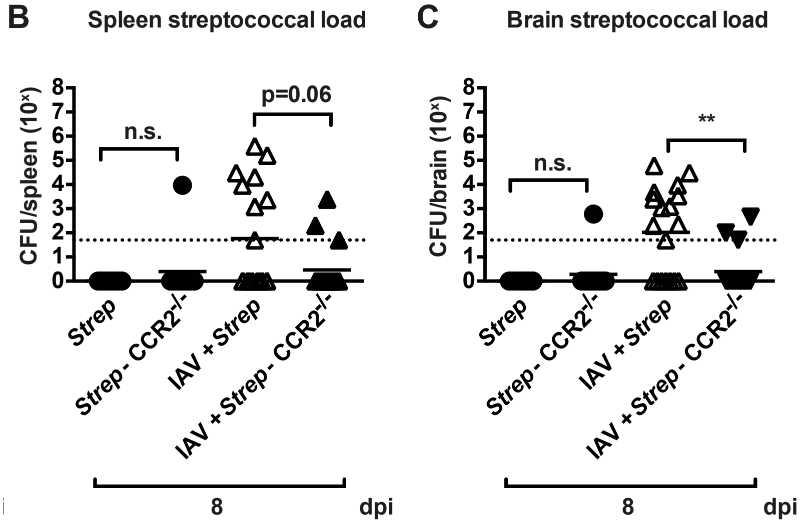

CFU/ spleen	Number of mice: *Strep*	*Strep* -CCR2 -/-	IAV + *Strep*	IAV + *Strep* -CCR2 -/-
>10⁴	0	0	4	0
50-10⁴	0	1	4	3
<50	9	9	10	13

CFU/ brain	Number of mice: *Strep*	*Strep* -CCR2 -/-	IAV + *Strep*	IAV + *Strep* -CCR2 -/-
>10⁴	0	0	2	0
50-10⁴	0	1	9	3
<50	9	9	7	13

To further demonstrate that CCR2 plays a role in coinfection in this model, a neutralizing Ab should be used. WT and CCR2 KO littermates were not used in Fig. 2. Moreover, this strategy might give additional information (e.g. treatment at early and later stage of influenza, as in Fig. 3D).

Concerning the use of littermates, as mentioned above, the attempt of generating enough littermates in a badly breeding line which we presently only have as a homozygous mice will be a major logistical effort and simply go beyond what is feasible in our mouse facility. We offer to perform microsatellite analysis to make sure the genetic similarity between CCR2 KO and B6 wt control mice that we use.
We agree that the use of a blocking mAb would be useful, in particular to study time course aspects. There is currently no efficient and specific depletion protocol for inflammatory monocytes, hence the widespread use of CCR2$^{-/-}$ mice. We attempted to use clodronate-liposomes but were not satisfied with their nonspecific toxic and depleting effect, we tried anti-Ly6C mAbs which do not deplete well, and we used anti-CD11b and anti-Gr-1 (the latter targeting Ly6C on monocytes and Ly6G on neutrophils) which both

deplete not only monocytes but also neutrophils, leading to an overall increased susceptibility which confirms to us the overriding importance of neutrophils in coinfection. We also used neutralising antibody as per referee 3's suggestion. We found mAbs against CCL2 (the main but not only CCR2 ligand) to be ineffective in blocking monocyte recruitment into the lung, suggesting that other CCR2 ligands can stand in for CCL2. This allowed us to ameliorate inflammatory-monocyte mediated damage at different periods during infection. We therefore have exhausted all strategies known to us that could remove monocytes, and they all were non-specific, inefficient or associated with toxicity. Blockade of TRAIL allowed us to partially circumvent this problem, by blocking an inflammatory monocyte effector function rather than depletion of the cells themselves.

Concerning the gating strategy shown in Fig. E2, the CD11b/CD11c labeling is not very discriminative (it is difficult to visualize CD11c-positive dendritic cells on the dot plot). The authors should use an anti-MHC class II Ab do make sure that there is no DCs in the inflammatory monocytes population. The authors might also use an anti-Siglec F Ab to label alveolar macrophages. What is the percentage of CCR2-positive cells within the inflammatory monocytes population?

We are grateful for these suggestions and can do more detailed stainings in wt and CCR2 KO mice to establish whether the lung recruitment defect is specific for monocytes, and we can show how many monocytes express CCR2 as far as commercial mAbs will allow this. Using our gating strategy, we find a complete lack of lung-recruited inflammatory monocytes in CCR2 KO mice, strongly suggesting that cells with this phenotype rely on CCR2 for recruitment and hence express it.

Judging by Fig. 3A (left panels), approximately 50% of TRAIL-positive cells are inflammatory monocytes. Do the authors know the nature of other TRAIL-positive cells? A more complete analysis should be done. This is important since the effect is probably not fully mediated by inflammatory monocytes. It might be interesting to show that TRAIL expression on inflammatory monocytes is involved in epithelial cell (as well as other cells) apoptosis. Do the authors have access to conditional knock-out mice? Usually, the anti-CD64 Ab is used to label inflammatory monocytes (Langlet et al. 2012). What is the percentage of CD64-positive "inflammatory monocytes" in flu infected mice?

We have neither CCR2 fl/fl nor TRAIL fl/fl mice to do such experiments. The referee is correct in saying that monocytes represent the majority but not totality of TRAIL positive cells, and we will perform detailed FACS to understand the distribution of other cell types expressing TRAIL.

In Fig. 4, depletion of neutrophils should be shown. Neutrophil depletion has no effect in mice only infected with Sp. I guess this is due to the low infectivity rate of D39 and that macrophages are implicated in this setting.

We can include the neutrophil depletion as shown below. In the stain to identify neutrophils shown below, Ly6G was not used again, to avoid mAb competition and false depletion rates. We agree that the low dose bacterium alone is inefficient at colonising and presumably been taken care of by alveolar macrophages.

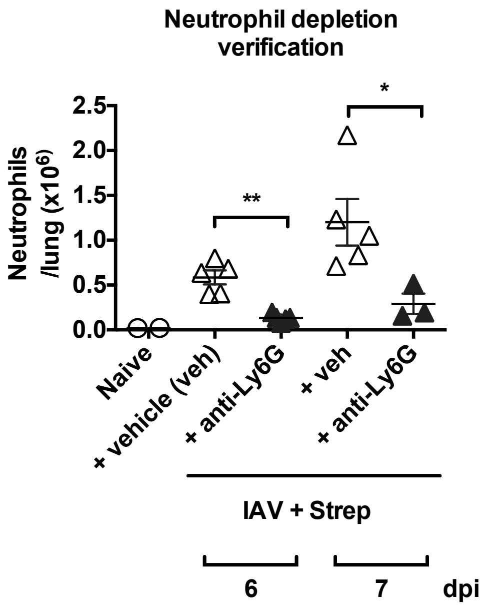

It might be interesting to determine the source of TNFa.

We show in fig. 4 of the manuscript that restimulated neutrophils from coinfected mice can make TNFa, but we don't know whether they are the principal source. We feel that answering this question would go beyond the scope of the present study.

To conclude, this is an interesting paper and the model used by the authors is interesting as it might mimic mild influenza/bacterial superinfection (although this can be debated; e.g. the high dose of bacteria used). The idea to separate events involved in bacterial colonization versus bacterial outgrowth is also well appreciated. Additional work is however needed to improve the quality of the manuscript.

We are grateful for the appreciation shown by the referee and hope that the proposed additional data and experiments here will help convince editors and referees of the solidity and novelty of our data.

1st Editorial Decision 14 April 2015

I have now heard back from referees 2 and 3, with whom I consulted regarding your plans for revision. Both referees and I agree that a manuscript revised along the lines you suggest could be a good candidate for publication in EMBO reports. I would like to therefore invite revision of your study, which will be sent back to referees 2 and 3 for their comments.

Regarding the points that you left open in your revision plan, although I appreciate the difficulty in achieving statistical significance with your clinically-relevant, mild infection system, we do feel that we cannot compromise here. Therefore, please do provide additional data in response to referee 1's point 1 and 3 (along the lines you suggest). In addition, please assess monocytes in circulation in response to referee 2's point 1 and include the scheme as a last figure. On the other hand, it would not be necessary to analyze mice at days 9 and 10 post infection (but rather more clearly dicuss the biological significance of your data) and I agree that identifying the source of TNFa would be beyond the scope of the present work. Please address all other concerns as you have indicated in the response to referees.

Revised manuscripts must be submitted within three months of a request for revision unless previously discussed with the editor; they will otherwise be treated as new submissions. Your study will be considered as an article, so please note that all of the materials and methods will need to be included in the main manuscript and Results and Discussion should be separate sections of the manuscript.

I look forward to receiving your revised study when it is ready. Please contact me if I can be of any help during the revision process.

1st Revision - authors' response 05 June 2015

1. Referee's comments addressed and our revisions and replies

Referee #1:

This manuscript presents three independent observations: 1) CCR2-mediated TRAIL+ monocyte recruitment is detrimental to the host; 2) neutrophils contribute to resistance against secondary bacterial infection; and 3) TNF-alpha is also protective during coinfection.

Although the reviewer classifies these observations as independent, we feel that presented together they provide a clear narrative of successive events in coinfection.

1. Fig 1 essentially repeats what others have consistently seen during influenza - pneumococcus co-infection. It is not obvious why it is felt that those findings need to be shown again in this manuscript.

As outlined, we have now repeated and pooled experiments to reach sufficient statistical power for all aspects of low-dose coinfection, and have replaced the entire figure 1 with a detailed characterisation of low-dose coinfection. Given the wide spread of data (e.g. lung bacterial load), we have moved the stratification of dying and recovering mice into this figure 1 as well, to show that bacterial loads are predictors of death versus recovery. As predicted, all parameters show the same type of changes as in high-dose coinfection, which is now shown in EV1 and EV2.

2. It has already been demonstrated by others that in the absence of CCR2 or TRAIL there is decreased inflammation from influenza virus infection. Like the current study, the previous papers reported no significant effect of CCR2 or TRAIL on influenza viral titers. If CCR2 KO mice and mice treated with anti-TRAIL antibody show diminished inflammation as already reported, decreased mortality and morbidity following bacterial co-infection as seen here, is to be expected.

We made a comprehensive reply to this point in our initial rebuttal, and now discuss this point more clearly in our manuscript. As agreed with the editor, no further action was required to address this point.

From our initial rebuttal: here Referee 1 argues that as the role of TRAIL and CCR2- dependent monocytes in causing damage and being on balance harmful has been previously investigated in severe influenza, that it can be implied that they will perform a similar function in coinfection. There are several points to be made which address this:

1.Coinfection is a clinically relevant, distinct disease context from influenza alone, and therefore the role of TRAIL and CCR2-dependent monocytes in coinfection merits separate investigation, and results are to be considered novel.

2.The net effect of CCR2-dependent monocytes and TRAIL as protective or harmful in influenza has not been conclusively established. Some studies report that they cause damage and are net harmful (e.g. Herold/Lohmeyer, J Exp Med, 2008).

However, others show a protective or harmful role depending on influenza severity (e.g. Aldridge/Thomas, PNAS, 2008). Also, these papers use highly severe or lethal flu models (80% mortality in Herold, 50-90% mortality in Aldridge, depending on the model), while flu in our model does not cause mortality, and no differences are found in single infections between wt and CCR2 KO mice. The referee points out correctly that "Like the current study, the previous papers reported no significant effect of CCR2 or TRAIL on influenza viral titers", but fails to report that there were massive changes in weight loss and mortality in the severe influenza infections, which we don't find in our single influenza infection (mortality is reported in fig. 3C, weight loss and clinical scores can be added for single and for coinfections). It is therefore not correct to simply extrapolate from published data to what we report here.

3.The second pathogen in coinfection - S. pneumoniae - must also be considered; as in influenza, there is not a clear consensus on their role. It has been reported in many studies of more severe S. pneumoniae infections that TRAIL and CCR2 are protective (Davis/Weiser, JCI, 2011; Winter/Maus JI 2007, 2009; Steinwede/Maus, J Exp Med, 2012), again indicating that single infection data does not predict coinfection outcome; however, other studies have found no effect in S. pneumoniae infection of the brain (Mildner/Prinz, J Imm, 2008).

4.Given the contradicting results found in influenza and S. pneumoniae infections, it is neither trivial nor in fact possible to extrapolate the role of TRAIL and CCR2- dependent monocytes from single infections to coinfection.

3.Was TRAIL expression analyzed on NK and T cells? It has been shown that influenza infection upregulates TRAIL expression on both NK and T cells (JIV, Ishikawa et al, 2005; JI, Brincks et al, 2008). Moreover, CD8+ T cells are known to be the primary effector cells responsible for the killing of flu infected epithelial cells, so even in the absence of TRIAL+ monocytes in CCR2-/- mice, significant killing of virally infected epithelial cells will still occur (and in fact, it does occur as shown in Fig 2F), yet CCR2 deficient mice show decreased susceptibility to secondary bacterial infection.

We have now repeated experiments and vastly extended the range of cell subsets on which we have assessed TRAIL expression, and have therefore extended figure 3A massively: As mentioned above, we do not find increased TRAIL expression or in fact appreciable numbers of TRAIL expressing cells among pDCs, CD8 T cells or NK cells. We have also refined the subsetting of monocytes, monocyte-derived DCs and conventional DCs, and alveolar and interstitial

© European Molecular Biology Organization

macrophages (gating strategy shown in EV3) and include these new subsets in fig. 3A. As requested by referee 2, we have also stained for CCR2 (see EV4) and find that three parameters are closely associated: CCR2 expression, loss of cell recruitment in CCR2-/- mice, and TRAIL expression. This fits perfectly with comparable improvement of coinfection severity in CCR2-/- mice and by TRAIL blockade.

Is this because there is a threshold effect of epithelial cell death for the increased susceptibility to secondary bacterial infection and this threshold is not reached in the absence of TRAIL+ monocytes in CCR2 deficient mice? It would be informative to quantitatively analyze by flow cytometry the absolute numbers of apoptotic/necrotic epithelial cells in WT and CCR2-/- mice following flu infection. If the authors are correct, the numbers of apoptotic/necrotic epithelial cell should be reduced in CCR2-/- deficient mice.

We agree that increased epithelial apoptosis is expected, but as anticipated, this is difficult to measure: we attach below our results obtained by FACS analysis of Annexin V- positive epithelial cells at day 5 post infection with influenza, and as expected we find reduced apoptosis in CCR2-/- mice compared to wild-type at the point of. We find however that despite extensive assay optimisation, a base line of 10% apoptotic epithelia is detected in naïve mice, which does not correspond to the reported extremely slow turnover of lung epithelia. We therefore think that while we can detect the trend that confirms our hypothesis, epithelial cell disruption during cell preparation for FACS causes a background level of apoptosis that is too high to allow for meaningful quantitative data on subtle differences.

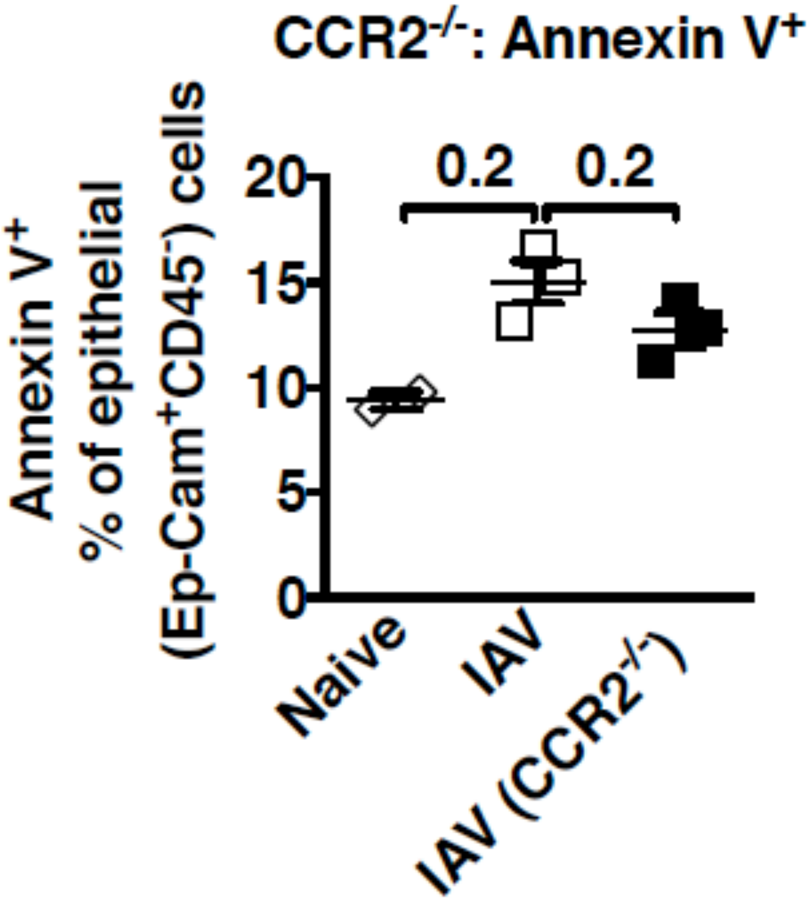

4. What is the influence of TRAIL and CCR2 on expression of types 1 and 2 interferon? As stated above, T and NK cells express TRAIL, these cells are a major source of IFN expression, and the IFNs have been shown to be the primary mediators of co-infection susceptibility.

It was agreed with the editor that no action has to be taken to reply to this concern.

5. Figs. 4-5, dealing with TNF-alpha and neutrophils are disconnected from the rest of the manuscript. It has been previously reported that TNF and neutrophils are required for protection against pneumococcal infection. The authors state that in the current study, they were only required for protection from co-infection. There is no further explanation but it is highly likely that the single bacterial infection in the current study was at such a low dose that the mice were fully protected from death by alveolar macrophage-mediated clearance and additional neutrophil-mediated protection was not required. Only during co-infection when there was large bacterial outgrowth would these innate mediators of protection be required. Thus, the explanation for the authors' observations is relatively trivial.

It was agreed with the editor that no action has to be taken to reply to this concern. We also addressed several of these points in our initial rebuttal (shown below), and also discuss these points in our revised manuscript:

From our initial rebuttal: there are several points to be addressed here:

1.Referee 1 states "TNF-alpha and neutrophils are disconnected to the rest of the manuscript". On the contrary, we feel that including this information provides a narrative of disease progression throughout coinfection, starting with upstream causes and progressing to downstream effects. It also demonstrates how different effector arms of the innate immune response are harmful at one stage of a complex infection (i.e. CCR2-dependent damage early) but protective later (i.e. neutrophils and TNF-α).

2.Referee 1 states "neutrophils are required for protection against pneumococcal infection". Although neutrophils are well established as protective in other bacterial infections, their role in S. pneumoniae has not been as clearly defined as commonly supposed. For example, neutrophil depletion using the non-specific mAb anti-Gr-1 exacerbates bacterial loads in some cases (Sun/Metzger, Inf Imm, 2007) but reduces it in others (Marks/Pirofski, Inf Imm, 2007), or has no effect (Stegemann/Gunzer, Plos One, 2009), or neutrophil function was proposed to be blocked by influenza coinfection (McNamee & Harmsen, IaI, 2006), which we do not find in our mild infection setting. Therefore the role of neutrophils in coinfection cannot be easily implied from their role in single S. pneumoniae infection.

3.We completely agree with the referee that neutrophils are not required here in single bacterial infection because this infection is mild. We however disagree with referee 1's statement that the role of neutrophils as protective in coinfection is a trivial induction from their role in single infection. There is a lack of consensus over the role of neutrophils in coinfection, which our model addresses. One study implies (although does not directly confirm through depletion studies) inadequate neutrophil recruitment is a cause of coinfection susceptibility (Shahangian/Deng, JCI, 2009), while another study shows no effect of neutrophil depletion in lethal coinfection (Damjanovic/Xing, Am J Path, 2013).

4.Neutrophils are known to be crucial effector cells in fulminant and chronic lung inflammation as characterised by acute respiratory distress syndrome (ARDS) or chronic obstructive pulmonary disease (COPD) (e.g. Ichikawa/Imai, AJRCCM 2013; reviewed in Short/Kuiken, Lancet 2014), and it is therefore a plausible hypothesis that the high levels of neutrophils and TNF-α may cause damage during the bacterial phase of coinfection. However, we demonstrate that the net effect is

protective. This is highly relevant in a study such as ours focusing on the role of immune-mediated damage in coinfection.

5.Although TNF-α has been shown to be protective in S. pneumoniae infection (e.g. Takashima/Yamaguchi, Inf Imm, 1997), this may be dependent on disease context (Kirby/Kaye, J Inf Dis, 2005). Furthermore it has been shown to have a harmful net effect in influenza infection (e.g. Hussell/Openshaw, Eur J Imm, 2001). Therefore showing it is protective in coinfection is a novel and clinically relevant observation; particularly as this may inform potential therapies.

6.The low pathogenicity of the single S. pneumoniae infection used in our system is not a flaw in the model, but a crucial element of it - we wanted to perform interventions in coinfection without perturbing the single infections greatly, as this would make interpreting results difficult. This required single infections of mild to moderate pathogenicity.

6.In the results section, FigE6A (page 13) is presented in the text before FigE5B,C and D. Reference to the figures should appear in the main text in numerical order.

We have now substantially reworked and renumbered all figures and they now follow the text in numerical order.

Referee #2:

Comments for the authors:

The authors of manuscript: "TRAIL+ monocytes induce lung damage increasing susceptibility to influenza-S. pneumoniae coinfection", present some very interesting results that evaluate the balance of pathogenesis or protection in the context of a coinfection. Specifically, the authors have identified the TRAIL+ monocytes as key cells that influence coinfection outcomes, based on whether they are allowed access to the lungs early during the viral phase of infection. This TRAIL-mediated lung damage is critical for allowing neutrophils, under the control of TNF- to limit bacterial outgrowth from the lungs. This study includes a large set of results that lead the authors to their conclusions. However, as presented, I do have some points of concern that I would like the authors to consider.
General Comments:

1. The data presented allow the authors to tell a very nice story, especially as it relates to the early vs. late anti-TRAIL therapy during a coinfection. I am also very interested in the pathogenesis vs. protection components of these studies. However, I am concerned with the interpretation of the results as it relates to CCR2-mediated recruitment of cells into the lungs. Since the lethal aspect of the coinfection was linked to the outgrowth of bacteria from the lungs, could another interpretation of the CCR2-/- data be that the effector cells remain in the periphery where they could more effectively eliminate the pathogen without the detrimental effects of inflammation within the lung? Maybe preventing TRAIL+ monocytes from entering the lung could be beneficial for the host's ability to handle the secondary bacterial invader. If the authors could comment on this aspect of their model, it would be appreciated.

As agreed with the editor, we have assessed this question and include the new data in fig. 2J: we find no monocytes in the blood of influenza infected mice at 5dpi. As this is the time point of coinfection, we can exclude the possibility that high numbers of monocytes are ready to deal with incoming bacteria in the blood. While a bar chart is included in the manuscript, we show here an original FACS plot for illustration:

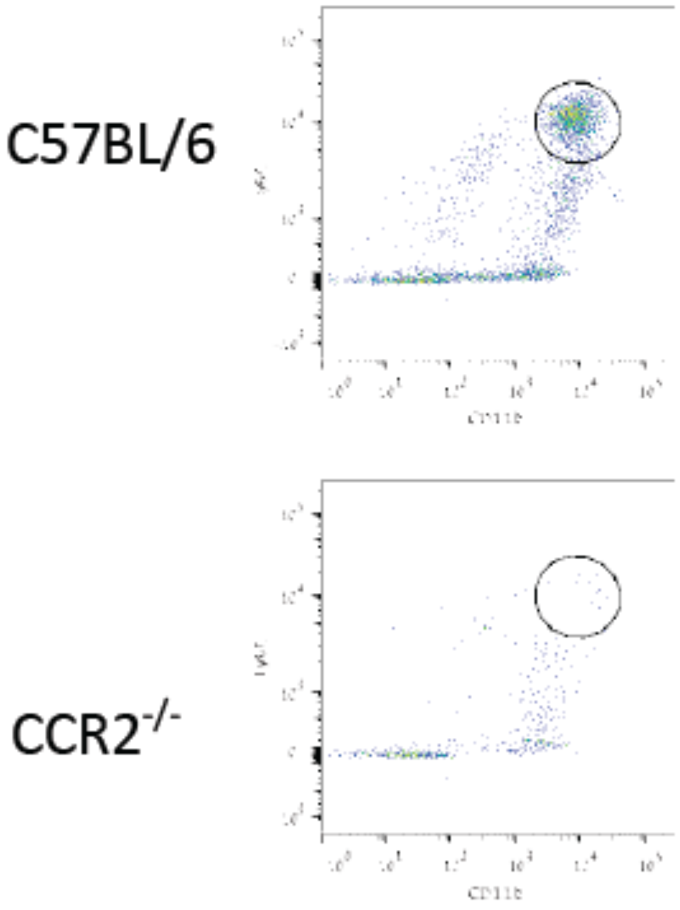

Blood from 5dpi influenza infected wild-type and CCR2 KO mice, CD11b/Ly6C stain to identify blood monocytes.

2. Some of the statistically significant differences reported did not appear to be biologically significant. This is most notable in Figure 4E where neutrophil depletion did not greatly affect bacteria within the lung. If the authors want to put forth the argument that increased survival in this model (Figure 4D) is due to more rapid clearance in the presence of neutrophils, then data from Day 9 and/or Day 10 should also be shown.

As discussed and agreed with the editor regarding the ethical and practical issues of sampling at 9 and 10dpi (many mice reach humane endpoint at day 8, and therefore any sample would be of the surviving mice, which would likely give a bias to a low bacterial load and confound the experiment), later sampling was not performed.

The graph referenced here is now figure 4G, as we have added more neutrophil data. We now explain this difference more clearly, emphasising (on page 17) that although the data shows a large spread (as is expected in low dose coinfection) depletion of neutrophils increases mean bacterial load more than 140-fold, which is likely highly biologically significant given the tight association between high bacterial loads and mortality shown in figure 1.

3. I would like to see a visual model added to the manuscript that directly shows the interpretation of the results that the authors present. At this time, I was unclear as to how the authors envision the

three factors studies (CCR2, TRAIL, and TNF-) work together to prevent death after coinfection. This was particularly difficult to visualize since the timing of anti-TRAIL treatment affected the outcomes, and this was based on whether the anti-TRAIL was delivered early (during the viral phase) or late (during the bacterial phase) of the infection.

As mentioned previously, we have now included a scheme to visualise our conclusions as figure 6.

4. I was less enthusiastic about the studies performed with purified neutrophils from the lungs of mice (Figure 4A-C). In particular, I think there are additional aspects of neutrophil function that need to be evaluated in the actual animal, rather than through removal of cells from the lungs, and evaluation in culture. It seems that the lack of a difference in neutrophil function that the authors report could be due to the fact that these cells were removed from the environment of the infected lung. I would prefer to see an attempt to characterize the neutrophils within the lung environment. This could be done by looking at NET formation, myeloperoxidase, defensins, lactoferrin, gelatinase/MMP9, and/or phagocytic neutrophils present in tissue sections (with immunofluorescent staining where appropriate).

We agree that harvesting neutrophils from the lung during infection may bias the selection towards neutrophils that have recently migrated to the lung or be attached to the lung endothelium.

Therefore we have now performed extensive histological analysis to confirm that neutrophils are functional in vivo during coinfection, and include this data in figure EV 5. In EV 5A, we show by confocal microscopy that bacterial capsular material (green) is found inside MPO positive neutrophils (magenta, with characteristic nuclei) as well as inside MPO negative phagocytic cells, presumably monocytes or macrophages. In coinfected lungs, the high number of MPO positive neutrophils that stain positive for bacteria clearly indicate that neutrophils take part in bacterial elimination by phagocytosis and that they are not impaired in this function in coinfection. We have also quantified the massive increase in MPO activity in coinfected airways by an ex vivo colorimetric assay and have included this new data as figure 4D. Figure EV 5B shows that NET formation is not a central mechanism of pneumococcus elimination in coinfection, as no extended areas with staining for the NET constituent citrullinated Histone H3 are found, in contrast to our positive control of Candida infected lungs. This is in line with our experiments of NET formation performed on ex vivo purified NPhs (fig. 4C): the ability of NET formation is retained by the purified neutrophils, but only Candida, not pneumococcus can trigger NET formation in vitro, and is in line with recent literature regarding this (Branzk et al. Nat.Imm. 2014, 1017-25).

5. Similar to comment 4, I was curious how the mice in the Ly6G-treated group that were infected with Strep alone (Figure 4D) performed within the clinical score evaluation (Figure E1B). Since there were scores for the Strep alone mice in the figure presented, does the absence of neutrophils in these mice increase the illness observed?

We include clinical scores for the Ly6G-treated mice here:

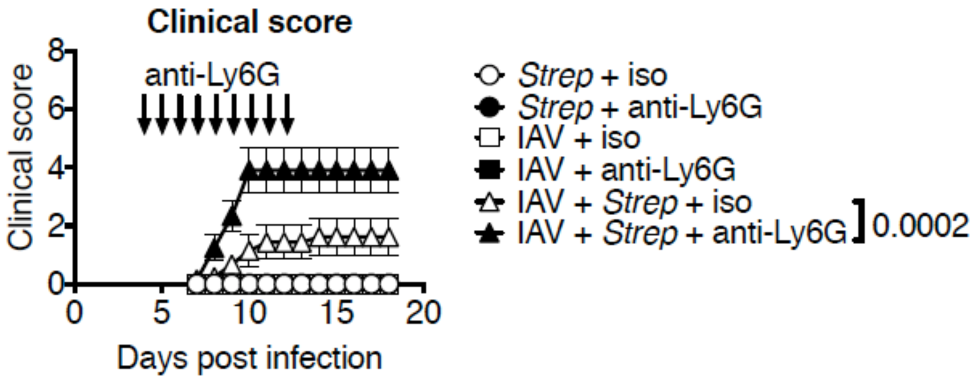

Since clinical scores are zero in single Strep-infected wild-type and Ly6G-depleted mice we chose to show this data here to the referees but to not include it in the paper, but we are willing to include this if required.

Specific Comments:

1. In the Introduction, the authors state that influenza virus infections are frequently complicated by secondary bacterial coinfections. It seems to me that this could be re- worded to state that deaths after influenza virus infections are frequently due to complications associated with secondary bacterial infections.

Thank you for the helpful suggestion, this has now been reworded on page 4.

2. In the third paragraph of the Introduction (Page 5, lines 9-11), I had a tough time understanding the point of this statement by the authors. Please clarify this statement.

This has now been reworded on page 5.

3. In the Results section (Page 9, line 7), the panel referenced (Figure E1B) shows that infection with S. pneumoniae alone shows a minimal clinical sign, rather than no clinical sign, and the authors should mention what was observed that led to the assigned scores given.

The clinical signs observed in this group (now figure EV 1C) have now been outlined on pages 9-10.

Referee #3:

In this manuscript, the authors developed a coinfection (IAV/S. pneumoniae) model of moderate severity to analyze mechanisms leading to bacterial colonization and bacterial outgrowth. The originality of the paper is to "separate" these two events and to mimic situation observed during regular influenza seasons (mild influenza). In this experimental system, the authors show (i) that CCR2 (probably by recruiting inflammatory monocytes) exacerbates susceptibility to bacterial infection by promoting tissue damage (through TRAIL expression) and (ii) that neutrophils protect against bacterial outgrowth in IAV-experienced animals, possibly through TNFa release. The subject is of great interest because clinically relevant, the model described is well controlled and the manuscript adds new information in the field. However, this manuscript suffers from a lack of mechanistic insights explaining in more details the role of inflammatory monocytes and neutrophils in bacterial superinfection.

It should be noted here although we show in vitro that restimulated neutrophils are functionally capable of producing TNF-α, we do not state that they are the main source of TNF-α in vivo, although this is possible. A full functional analysis of neutrophil action is now included in form of new data in EV 5 and fig. 4D.

Below are other issues that, if addressed, might improve the quality of the manuscript.

The first part dedicated to the acute severity coinfection model (high dose) is too long and too descriptive. This part does not really provide new information. Moreover, the dose used (2x10e7 bacteria) does not really correspond to a "physiological" dose. This is enormous. For the rest of the study, the authors concentrate on the moderate ("low dose") severity coinfection model. Here too,

the dose (2x10e5) is quite important (D39 has a low infectivity potential in the mouse system) and might raise concern about the significance of the data shown in the manuscript.

As pointed out in reply to referee 1's point 1 and in agreement with the editor, we have now fully replaced the old figure 1 with a new one characterising low dose coinfection, and observed similar results (as expected, with greater spread within groups) to previous high dose profiling. The original data on high-dose coinfection has been moved to figure EV 1 and 2.

In Fig. 1B, the authors claimed (page 9) that viruses are cleared but this is not the case (the viral load is just reduced). This should be reworded.

This has now been reworded on page 10 to "virus titers were unaffected by the bacterial superinfection".

In Fig. 2 (page 11), there is an improved control of bacterial outgrowth and survival in CCR2 deficient animals. Is it associated with reduced recruitment of inflammatory monocytes in the lungs?

We are not sure if this question refers to single bacterial infection or to coinfection. For single infection: We essentially find no monocyte recruitment above naïve baseline in single pneumococcus infection (new fig. 2I) in wild-type mice, and the rare cases of bacterial outgrowth in a small number of animals do not translate into statistical significance (as shown in Figure 2B and,2C). If the coinfection is referred to, then the answer is yes: there is massive loss in monocyte recruitment on both 5 and 7dpi, as shown in Figure 2H and the new data in 2I, and this is strongly associated with improved bacterial control.

The authors claim that inflammatory monocytes are involved in CCR2-mediated bacterial superinfection but this is not shown.

We have confirmed that CCR2 deficiency leads to an almost total absence of monocytes and monocyte derived cell types in the lung both at the point of coinfection (5dpi) and following bacterial infection (7dpi). This data is now included in the manuscript as Figures 2H and I.

The authors state that the numbers of CFU are decreased in spleen and brain (page 12) but this is not shown.

The data for brain is shown below, and we have included the spleen data in the manuscript as new fig. 2C. While we have omitted reference to brain in the paper, we are happy to include this if preferred by the referee.

© European Molecular Biology Organization

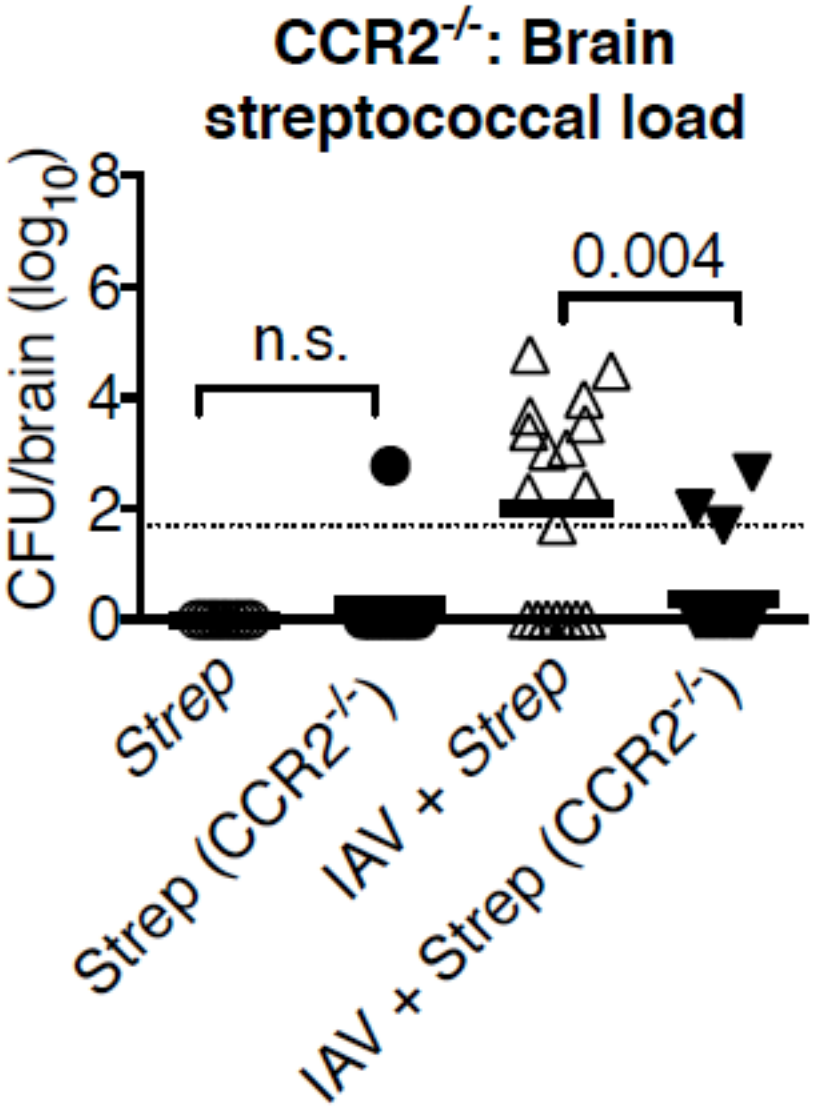

To further demonstrate that CCR2 plays a role in coinfection in this model, a neutralizing Ab should be used. WT and CCR2 KO littermates were not used in Fig. 2. Moreover, this strategy might give additional information (e.g. treatment at early and later stage of influenza, as in Fig. 3D).

We agree that the use of a neutralising antibody would be useful, in particular to study timing aspects. There is currently no efficient and specific depletion protocol for inflammatory monocytes, hence the widespread use of CCR2-/- mice. We attempted to use clodronate-liposomes but were not satisfied with their nonspecific toxic and depleting effect, we tried anti-Ly6C which did not deplete well, and we used anti-CD11b and anti-Gr- 1 (the latter targeting Ly6C on monocytes and Ly6G on neutrophils) which both deplete not only monocytes but also neutrophils, leading to an overall increase in susceptibility which confirms to us the overriding importance of neutrophils in coinfection. We found anti-CCL2 (the main but not only CCR2 ligand) to be ineffective in blocking monocyte recruitment into the lung, suggesting that other CCR2 ligands can stand in for CCL2. We therefore have exhausted depletion strategies known to us that could remove monocytes, and they all were non-specific, inefficient or associated with toxicity. Blockade of TRAIL allowed us to partially circumvent this problem, by blocking an inflammatory monocyte

© European Molecular Biology Organization

effector function rather than depletion of the cells themselves. It also allowed us to administer treatments at different timings to dissect the timing of events in coinfection. Concerning the use of littermates, as mentioned previously, the attempt of generating enough littermates in a badly breeding line which we presently only have as a homozygous mice will be a major logistical effort and simply go beyond what is feasible in our mouse facility.

Concerning the gating strategy shown in Fig. E2, the CD11b/CD11c labeling is not very discriminative (it is difficult to visualize CD11c-positive dendritic cells on the dot plot). The authors should use an anti-MHC class II Ab do make sure that there is no DCs in the inflammatory monocytes population. The authors might also use an anti-Siglec F Ab to label alveolar macrophages. What is the percentage of CCR2-positive cells within the inflammatory monocytes population?

We have now greatly extended the staining and subsetting and follow the guidelines suggested by the referee and shown in Langlet et al. and Misharin et al. In particular, we include MHC II, Siglec F, CD24 and CD64 in our stainings to distinguish between inflammatory monocytes (IM, Siglec F- CD11b+ MHC II- Ly6C+ Ly6G- CD64+), monocyte- derived DCs (Mono d. DC, Siglec F- CD11b+ MHC II+ Ly6C+ Ly6G- CD64+ CD11c+) and interstitial macrophages (Inter. Mac, Siglec F- CD11b+ MHC II+ Ly6C+ Ly6G- CD64+ CD11c- CD24-) which are all different to alveolar macrophages (AM, Siglec F+ CD11b int MHC II int Ly6C+ Ly6G- CD64+ CD11c+). Very similar to what is shown in Langlet for muscle, we find that IMs, Mono d. DCs and Inter. Macs are not recruited into the infected lung in CCR2-/- mice. Interestingly, two of these cell subsets also express high levels of CCR2 (Figure EV4), while monocyte derived DCs have lost CCR2 expression, suggesting that they were most likely recruited into the organ as monocytes. The same three populations of IMs, Mono d. DCs and Inter. Macs show the highest levels of TRAIL expression, and together they constitute the near-totality of TRAIL+ cells in the infected lung. In contrast, AM, conventional DCs, pDCs, NK and CD8 T cells are not TRAIL positive and do not depend on CCR2 for their recruitment. This data is now included as Figure EV4 and Figure 3A.

Judging by Fig. 3A (left panels), approximately 50% of TRAIL-positive cells are inflammatory monocytes. Do the authors know the nature of other TRAIL-positive cells? A more complete analysis should be done. This is important since the effect is probably not fully mediated by inflammatory monocytes. It might be interesting to show that TRAIL expression on inflammatory monocytes is involved in epithelial cell (as well as other cells) apoptosis. Do the authors have access to conditional knock-out mice? Usually, the anti-CD64 Ab is used to label inflammatory monocytes (Langlet et al. 2012). What is the percentage of CD64-positive "inflammatory monocytes" in flu infected mice?

As described above an extended analysis of myeloid and lymphoid subsets expressing TRAIL is now found in EV4 and fig. 3A. We think we have exhaustively characterized the TRAIL positive immune cell subsets in our system. Since we find a closely related group of myeloid immune cells absent in CCR2-/- lungs and TRAIL positive, we have extended the title of the paper accordingly to accommodate these important findings.

In Fig. 4, depletion of neutrophils should be shown. Neutrophil depletion has no effect in mice only infected with Sp. I guess this is due to the low infectivity rate of D39 and that macrophages are implicated in this setting.

We now include our verification of neutrophil depletion as Figure 4E. In the stain to identify neutrophils shown, Ly6G was not used, to avoid monoclonal antibody competition and false depletion rates. We agree that the low dose bacterium alone is inefficient at colonising and presumably been taken care of by the epithelial barrier and alveolar macrophages, hence the lack of effect of neutrophil depletion.

It might be interesting to determine the source of TNFa.

We show in Figure 4 of the manuscript that restimulated neutrophils from coinfected mice can make TNFa, but we don't know whether they are the principal source. As agreed with the editor, we have not pursued this question for inclusion in the present manuscript.

To conclude, this is an interesting paper and the model used by the authors is interesting as it might mimic mild influenza/bacterial superinfection (although this can be debated; e.g. the high dose of bacteria used). The idea to separate events involved in bacterial colonization versus bacterial outgrowth is also well appreciated. Additional work is however needed to improve the quality of the manuscript.

We are grateful for the appreciation shown by the editor and the referees and we were able to address all issues raised by the referees and endorsed by the editor. We hope that the proposed additional data and experiments here will convince editors and referees of the solidity and novelty of our study for publication in EMBO reports.

2nd Editorial Decision 01 July 2015

Thank you for your patience while we have reviewed your revised manuscript. As you will see from the reports below, the both referees are now positive about the study, although referee 1 (former referee 3) still has some issues. I believe, however, that at this stage these can all be addressed in writing and no further experimentation will be necessary prior to publication. They are, however, relevant points, so please do address them.

Given the overall positive evaluation, I am writing with an 'accept in principle' decision, which means that I will be happy to accept your manuscript for publication once you have modified the study as indicated above and attended a few minor formatting issues, as follows.

- Please add a statement in the "clinical scoring" section of the materials and methods to indicate that no blinding was performed when assessing the phenotypes.

After all remaining corrections have been attended to, you will receive an official decision letter from the journal accepting your manuscript for publication in the next available issue of EMBO reports. This letter will also include details of the further steps you need to take for the prompt inclusion of your manuscript in our next available issue.

REFEREE REPORTS:

Referee #1:

This paper is solid, very well controlled, straight to the point, and data support the conclusions. It adds new information in the field. Although the authors have addressed the major issues raised by the reviewers, below are comments that, if addressed, might improve the quality of the manuscript.

Major comments
An important aspect that needs further investigation, and which is not yet enough convincing, is the relationship between TRAIL-expressing cells, epithelial damage and bacterial susceptibility. In other words, the authors should show histological scoring of epithelial damage in isotype-treated versus anti-TRAIL Ab-treated animals. The authors show protein concentration and LDH concentration in the BAL, but this is not sufficient. Other parameters showing epithelial barrier functions and/or expression of genes associated with epithelial barrier functions would be informative. Determination of epithelial cell apoptosis might also be interesting although I understand this might raise technical problems. Having said that, the background is quite low in

Herold et al. (2008)'s paper published in J. Exp. Med (2008) (Fig. 4). The authors also mention altered ciliated functions as a mechanism driving bacterial superinfection. Can this be assessed for instance by measuring expression of genes associated with mucosal-ciliary functions?

Another point that needs further consideration is the gating strategy used to analyze TRAIL-expressing cells. The authors should be more cautious in their interpretation (MI) since F4/80 expression is not considered. Anti-CD68 might also be useful to characterize macrophages. Are all MI MHC class II positive (I am not sure)? Please precise if the analysis was performed in the BAL or whole lungs.
Along with the analysis of TRAIL-expressing cells, quantification of soluble TRAIL in the BAL fluids is also important to show (WT vs CCR2 KO mice).
TRAIL plays a negative role during severe influenza whilst it appears to play a minor, if any, role during mild (e.g. x31) infection (Herold et al. (2008). In Herold et al. (2008) (Fig. 7), TRAIL expression was not detected after infection with mild influenza whilst in the present paper, it is. This should be discussed (the authors also used x31 in the present manuscript).

Minor comments
The text could be shortened. The first part (Fig. 1) is too long. Is it crucial to mention bacterial RNA quantification (EV1J)? The discussion should be shortened.

The authors state page 18 that 1918 influenza was not pathogenic but in page 23 (line 6) they mention it was.

The authors mention in the text that "inflammatory monocytes induce TRAIL-mediated lung damage". Please be more caution since other cells (IM, MoDC, ..) could also do so. In the text (the title should also be modified), the term "inflammatory monocytes and related population" does not seem to be optimal. "TRAIL-expressing inflammatory myeloid cells" might be more appropriate.

Page 13, the authors mention a recruitment of pDCs and CD103+ DCs. Judging by Fig. 2H, there is no pDCs and CD103+ DCs in IAV-infected mice. Please reword.

The figures could be improved. For instance (Fig. 2), please replace "IAV" by "IAV (WT)". In Fig. 3, vehicle (veh) should be replaced by "isotype control". etc

Page 14, line 7: the reference "Mc Cullers 2014" is not appropriate here. The authors might add Hogner et al. Plos Path 2013.e1003188.

Referee #2:

I thank the authors for completely and adequately addressing my previous comments, and I have no further suggestions for this manuscript.

2nd Revision - authors' response 10 July 2015

Dear Editor and Referees,

We have now made all required changes as listed below:

Editor:

-Please add a statement in the "clinical scoring" section of the materials and methods to indicate that no blinding was performed when assessing the phenotypes.

Added now on page 25.

Referee #1:

This paper is solid, very well controlled, straight to the point, and data support the conclusions. It adds new information in the field. Although the authors have addressed the major issues raised by the reviewers, below are comments that, if addressed, might improve the quality of the manuscript.

Major comments

-An important aspect that needs further investigation, and which is not yet enough convincing, is the relationship between TRAIL-expressing cells, epithelial damage and bacterial susceptibility. In other words, the authors should show histological scoring of epithelial damage in isotype-treated versus anti- TRAIL Ab-treated animals. The authors show protein concentration and LDH concentration in the BAL, but this is not sufficient. Other parameters showing epithelial barrier functions and/or expression of genes associated with epithelial barrier functions would be informative. Determination of epithelial cell apoptosis might also be interesting although I understand this might raise technical problems. Having said that, the background is quite low in Herold et al. (2008)'s paper published in J. Exp. Med (2008) (Fig. 4).

While we agree that further damage parameters would help confirm our BAL LDH and protein results demonstrating that TRAIL blockade reduces lung damage, we think that the mild influenza infection we chose to use makes this very difficult: The main issue for our FACS apoptosis experiments that were included in the last round of revision is that tissue processing causes a background level of apoptosis giving a poor signal to noise ratio. The above cited experiments by Herold et al. use highly virulent PR8 and look at infections causing 80% mortality. In contrast, our single influenza infection is done with the less virulent X31 and causes less than 10% mortality. In both Herold et al. and our study, background is approximately 10% or slightly lower at baseline. However due to higher virulence Herold et al. detect up to 40% apoptosis in infected wild-type mice (as opposed to approximately 15% in our study), and therefore the signal-to-noise ratio is very different; hence more than a trend will be difficult to obtain. Similar considerations have to be made for histological scoring. To illustrate this, we include a TUNEL stain on day 5 of influenza infection: while we see a trend to lower apoptosis in infected CCR2-/- lungs, this difference is too subtle to render statistically significant without large sample numbers – we do however think this is biologically relevant.

© European Molecular Biology Organization

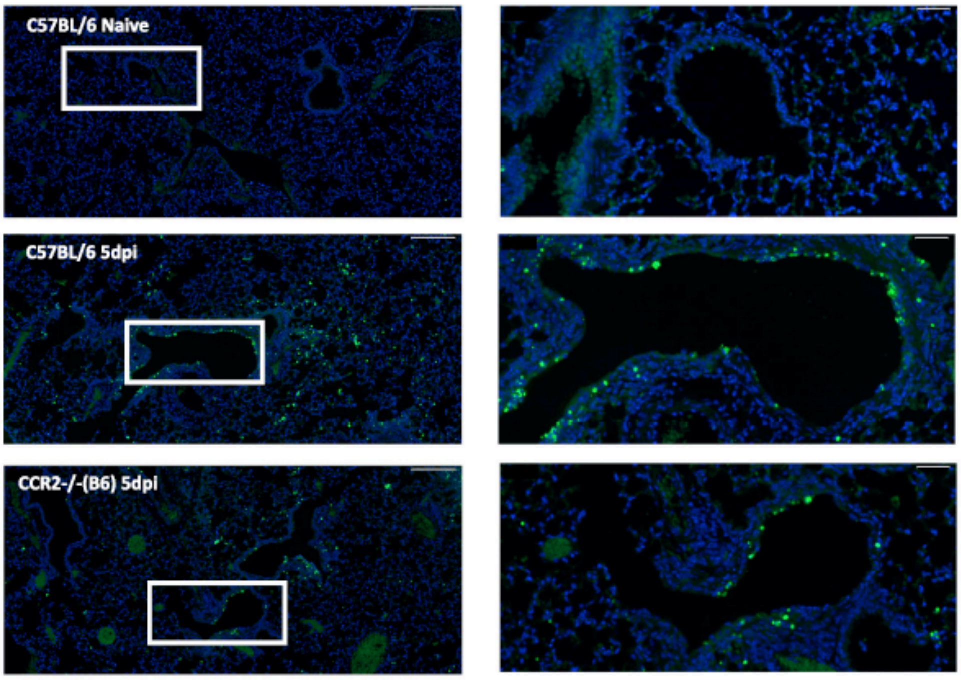

-The authors also mention altered ciliated functions as a mechanism driving bacterial superinfection. Can this be assessed for instance by measuring expression of genes associated with mucosal-ciliary functions?

This is not the mechanism under investigation in this study. The paper we cite looks at mucociliary velocity, which can be caused by disorganization or absence of cilia or ciliated cells and therefore may or may not be reflected in gene expression levels. In addition, the epithelial damage caused during influenza infection destroys both secretory and ciliated epithelial cells. For these two reasons, reduction of cilia-related genes is potentially difficult to interpret, especially if assessed in relation to reduction in other epithelial cell types.

- Another point that needs further consideration is the gating strategy used to analyze TRAIL-expressing cells. The authors should be more cautious in their interpretation (MI) since F4/80 expression is not considered. Anti-CD68 might also be useful to characterize macrophages. Are all MI MHC class II positive (I am not sure)? Please precise if the analysis was performed in the BAL or whole lungs.

It is difficult to reply to this point as we do not term any cell type as MI in the manuscript; we will assume the reviewer refers to Interstitial Macrophages and answer as such. Our definition of Interstitial Macrophages follows what has previously been published by Misharin et al. 2013, where all Interstitial Macrophages are all defined as MHCII+. Should the reviewer refer by MI to inflammatory monocytes, these are by our and others' definition MHC II negative as their MHC II positive counterparts are monocyte-derived DCs. Based on previous studies, particularly Misharin et al. 2013, Langlet et al. 2012 and our own research, we think we have performed a comprehensive analysis of CCR2 dependent myeloid populations in the infected lung. We do not feel that addition of F4/80 would improve subset definition, as F4/80 is not a good marker for macrophages in the lung, it is expressed at relatively low levels, and we do not need it to define Alveolar Macrophages which we unequivocally identify using Siglec F and CD11c, as done by

many groups. Similarly, our gating strategy would not benefit from inclusion of CD68, which according to Zaynagetdinov et al. 2013 has low expression levels on Interstitial Macrophages and is highly expressed on Alveolar Macs (already defined, see above). In the study mentioned, CD68 is also expressed on CD103+ DCs (CD68hi/F4/80- /CD11c+/Gr1-/CD103+/MHCIIhi) which we confirm do not express TRAIL, or depend on CCR2 for recruitment during influenza infection. The analysis was performed on whole lungs which had not had been lavaged, as now indicated in the legends to figures EV3 and EV4.

-Along with the analysis of TRAIL-expressing cells, quantification of soluble TRAIL in the BAL fluids is also important to show (WT vs CCR2 KO mice).

In past experiments, we were able to measure significant amounts of soluble TRAIL in supernatants from human cell culture, but not from comparable mouse cell culture or ex vivo. We found no soluble TRAIL in two experiments using BAL from wild-type and CCR2-/- mice. We do not know if this is a biological difference between humans and mice or if this reflects differences in the tools used to detect mouse and human soluble TRAIL. Given these limitations, we prefer to rely on the TRAIL expression levels we find on the surface of immune cells. If the referee wishes, we can include that we did not detect soluble TRAIL in BAL as data not shown, however we would prefer not to as we have not seen any positive signal for mouse soluble TRAIL.

TRAIL plays a negative role during severe influenza whilst it appears to play a minor, if any, role during mild (e.g. x31) infection (Herold et al. (2008). In Herold et al. (2008) (Fig. 7), TRAIL expression was not detected after infection with mild influenza whilst in the present paper, it is. This should be discussed (the authors also used x31 in the present manuscript).

We have discussed this important difference now on page 21 of the annotated manuscript. Infection severity is impossible to compare as Herold et al. characterize their infection as "non-lethal", without indicating maximal weight loss which would allow comparison to the pathogenicity in our infection model.

Minor comments

-The text could be shortened. The first part (Fig. 1) is too long. Is it crucial to mention bacterial RNA quantification (EV1J)?

We have slightly shortened the results part. EV1J is a helpful control to confirm that the rapid reduction in bacterial numbers seen in singly infected mice is a true reflection of infection efficiency, rather than due to a problem with inoculation, and therefore we feel this is an essential part of the paper.

The discussion should be shortened.

We have slightly shortened the discussion part.

The authors state page 18 that 1918 influenza was not pathogenic but in page 23 (line 6) they mention it was.

Apologies for a confusing statement on page 18 which has now been fixed.

The authors mention in the text that "inflammatory monocytes induce TRAIL-mediated lung damage". Please be more caution since other cells (IM, MoDC, ..) could also do so. In the text (the title should also be modified), the term "inflammatory monocytes and related population" does not seem to be optimal. "TRAIL-expressing inflammatory myeloid cells" might be more appropriate.

While we find the suggested term helpful, "inflammatory myeloid cells" (a term which could include neutrophils, eosinophils and other populations) is too generic to indicate the close relationship between the TRAIL-expressing populations we identify, as indicated by their CCR2

© European Molecular Biology Organization

dependency for recruitment. At several points in the text we clearly indicate that we refer to inflammatory monocytes and monocyte-related cells, including in the main title. The referee is correct that in one results section title we still refer only to monocytes, and we have fixed this (page 14), as we have fixed in the title of figure 2.

Page 13, the authors mention a recruitment of pDCs and CD103+ DCs. Judging by Fig. 2H, there is no pDCs and CD103+ DCs in IAV-infected mice. Please reword.

We have reworded from recruitment to numbers.

The figures could be improved. For instance (Fig. 2), please replace "IAV" by "IAV (WT)". In Fig. 3, vehicle (veh) should be replaced by "isotype control". etc

We have made genotype annotations clearer in fig. 2 and fig. 3.

Page 14, line 7: the reference "Mc Cullers 2014" is not appropriate here. The authors might add Hogner et al. Plos Path 2013.e1003188.

We have fixed this now.

Referee #2:

I thank the authors for completely and adequately addressing my previous comments, and I have no further suggestions for this manuscript.

Thanks!

3rd Editorial Decision 10 July 2015

I have now gone through your revised version and am very pleased to accept your manuscript for publication in the next available issue of EMBO reports.

Thanks for your contribution to EMBO reports and congratulations on a successful publication. Please consider us again in the future for your most exciting work.

© European Molecular Biology Organization
